# Defining Spirituality in Healthcare: A Systematic Review and Conceptual Framework

**DOI:** 10.3389/fpsyg.2021.756080

**Published:** 2021-11-18

**Authors:** Marina Aline de Brito Sena, Rodolfo Furlan Damiano, Giancarlo Lucchetti, Mario Fernando Prieto Peres

**Affiliations:** ^1^Instituto de Psiquiatria, Hospital das Clínicas HCFMUSP, Faculdade de Medicina, Universidade de São Paulo, São Paulo, Brazil; ^2^School of Medicine, Federal University of Juiz de Fora, Juiz de Fora, Brazil

**Keywords:** spirituality, religion, religion and psychology, religion and medicine, healthcare

## Abstract

**Objective:** To investigate the definitions of spirituality in the healthcare field, identifying its main dimensions and proposing a framework that operationalizes the understanding of this concept.

**Methods:** This is a systematic review following the PRISMA guideline (PROSPERO: CRD42021262091), searching for spirituality definitions published in scientific journals. Searches were carried out in PubMed (all articles listed up to October 2020) and in the reference lists of the articles found in the database, followed by selection under specific eligibility criteria.

**Results:** From a total of 493 articles, 166 were included in the final analysis, showing that there is a large body of scientific literature proposing and analyzing spirituality definitions. In these articles, 24 spirituality dimensions were found, most commonly related to the connectedness and meaning of life. Spirituality was presented as a human and individual aspect. These findings led us to construct a framework that represents spirituality as a quantifiable construct.

**Conclusions:** Understanding spirituality is an important aspect for healthcare research and clinical practice. This proposed framework may help to better understand the complexity of this topic, where advances are desirable, given the relevance it has acquired for integral health care.

## Introduction

Spirituality is a broad and complex concept which varies its understanding according to different cultural, religious and academic backgrounds (i.e., religious persons, scientists, or lay persons; Koenig, 2008; la Cour and Götke, 2012). In this context, there is a remarkable debate regarding the most accurate meaning, and regarding the possibility of having a single universal consensual definition for this concept (Peng-Keller, 2019). Some issues arise since the fact that spirituality is often linked and overlaps another important concepts, such as religion/religiosity and well-being/positive emotions (Hill et al., 2000).

Historically, the term spirituality was used to describe the practices of people who dedicated their lives into religious services or exemplify the teachings of their faith traditions (Koenig, 2008). Only in the last decades, spirituality has been detached from religiosity as a distinct construct, even though the scientific community still refers to this research field using the “dual” term religiosity/spirituality (R/S; Zinnbauer et al., 1997; Bauer and Johnson, 2019).

Research over the last decades has been growing substantially in the field of “Spirituality and Health,” showing a significant influence of spiritual and religious beliefs on both mental and physical health outcomes (Damiano et al., 2016), and approximately 30,000 articles have been published in this field of research from 1999 to 2013 in the PubMed database (Lucchetti and Lucchetti, 2014). In addition, the spiritual dimension has been proposed to be included in the multidimensional concept of “health,” as illustrated by discussions in the scope of the World Health Organization (WHO), which referred to the “*inclusion of a non-material or spiritual health dimension, making the concept come to be regarded as a dynamic health state - physical, mental, spiritual and social behavior*” (Grad, 2002; Dhar et al., 2011; Toniol, 2017).

This discussion is supported by a robust body of evidence suggesting a significant effect in physical, mental, and social health (Koenig, 2015; Zimmer et al., 2016; Mishra et al., 2017). Spirituality is generally related to diminished numbers of substance use, suicidal attempts and depression prevalence, less hospitalization, better coping with disease, better treatment adherence, and lower mortality rates (Moreira-Almeida et al., 2006; Guimarães and Avezum, 2007; Lucchetti et al., 2011). In addition to the clinical importance observed, patients want their doctors to address spirituality and most doctors and nurses consider important to integrate this aspect into their practice (Baetz et al., 2004). However, several barriers limit addressing R/S, including the lack of training by health professionals and the lack of clear defined concepts (Best et al., 2015; Menegatti-Chequini et al., 2019). In this context, the understanding of Spirituality becomes an important issue for research, clinical practice, and the training of health professionals (Lucchetti et al., 2012; Attard et al., 2019).

Despite the increasing use of the concept of spirituality among health researchers, there is no clear consensus about its definition (Chiu et al., 2004; Sessanna et al., 2007; Gall et al., 2011). This lack of standardized definition increases the potential for non-standardized constructs, creating pitfalls while comparing studies that use different criteria and instruments, especially in health-related researches. While for the social sciences there is no major concerns for the lack of an universal definition of spirituality, medical and health-related sciences need a structure and relative consensus, since most instruments attempt to quantify its intangible construct in order to evaluate its impact and propose health-related interventions (Macdonald and Friedman, 2002; Hill and Pargament, 2008).

Although a previous study has already used qualitative content analysis of the published literature (Elkins et al., 1988), in the last decades, the field of spirituality has considerably changed with a large number of publications and ongoing researches. Likewise, several articles were published with new definitions and concepts. Therefore, it is of urgent necessity to better clarify and disentangle the concepts of spirituality and religiosity, determining and understanding which dimensions of spirituality influence more positively health-related endpoints. In this sense, the present article aims to move forward on this discussion, presenting a systematic review of the spirituality concept for the healthcare field, identifying its main dimensions and proposing a framework that operationalizes the understanding of the term spirituality.

## Materials and Methods

This is a systematic review based on the PRISMA statement for reporting systematic reviews and meta-analyses (Page et al., 2021). The protocol was registered in the PROSPERO international prospective registry of systematic reviews (registration number: CRD42021262091).

### Eligibility Criteria

The following criteria were applied to include the studies in this review: articles that addressed the meaning, concept, or definition of spirituality (either new proposals of definitions in the healthcare area or operational definitions that analyzed pre-existent definitions in the literature). All articles (letters to the editor, editorials, opinion essays, observational studies) were included. No language or date restrictions were applied. The exclusion criteria were articles that were not available in full, articles not related to the definition of spirituality, and those that did not present a new concept or operational definition about spirituality.

### Search Strategy

The literature search was conducted using the PubMed database (all articles listed up to October 1, 2020), with the Boolean expression “*spirituality [title] AND (concept OR definition)*” and scanning reference lists of the included articles.

### Study Selection

The selection of studies was conducted in three stages:

**Stage 1:** All references on the PubMed database were screened using the Boolean expression described above; additional records were identified through the list of references of the articles obtained. Duplicates were excluded using the Endnote software. Eligibility was determined based on title and/or abstract. Articles that brought a new proposal of spirituality definition or analysis of definitions already existing were considered and included. All included articles on stage 1 proceeded to stage 2.

**Stage 2:** The articles were read in full, focusing on the eligibility criteria and on evaluating the characteristics of the article (authors, year of publication, number of citations, language) and of the definition (discursive or in topics, newly proposed, operational definition or citation). Articles that only cited pre-existing definitions were excluded, but their lists of references were used as a secondary source.

**Stage 3:** All definitions of spirituality found were analyzed, seeking to identify the dimensions they presented.

### Data Extraction and Analysis

All definitions of spirituality were analyzed looking for expressions or terms that could characterize a dimension. The conceptual dimensions were identified using expressions or terms that were repeated and/or carried a similar meaning among the different definitions, for example: the expressions “these dimensions of spirituality are applicable to all human beings” and “spirituality refers to a fundamental aspect of humanity” are part of different definitions of spirituality and have been classified as the “human dimension” simply (Anandarajah, 2008; Appleby et al., 2018). After identifying all dimensions, from all selected definitions, a second author looked up into each definition in order to confirm and unify all chosen dimensions. In this step, a score was established to quantify the use of a given term/expression, with each use corresponding to one point. The sum of the number of points was transformed into percentage, with 166 corresponding to 100%, since 166 was the total number of definitions analyzed. Terms that did not appear in at least 3 definitions were excluded, as they corresponded to less than 2% appearance in the definitions.

### Framework Development

The results led us to construct a framework, organizing the correlated dimensions in horizontal axes, representing spirituality in a visual structure (see the “Discussion” section below).

## Results

### Study Selection and Characteristics

**Figure 1:**
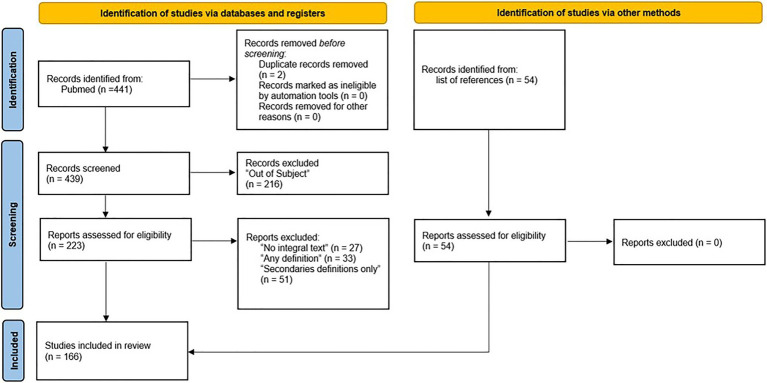
PRISMA search strategy used in the present study.

We found 441 articles in the PubMed query and 54 additional records were identified in secondary sources. After excluding duplicates, a total of 493 articles remained for the first screening. From them, 277 were accepted for full text reading. After full text reading, 111 articles were excluded, leaving a total of 166 articles, most of which in English, that were included in final analysis concerning spirituality definitions. Figure 1 summarizes the steps of the systematic review. Tables 1 and 2 presents, respectively, the most cited articles and books on November 3, 2020, according to Web of Science and Google Scholar.

**Table 1:** Most cited articles, according to Web of Science and Google Scholar Citations in November 3, 2020, found in the present study.

Ranking	Article	Year	No. Wos citations	No. Google Scholar citations
1	Hill, P. C., Pargament KI. Advances in the conceptualization and measurement of religion and spirituality. Implications for physical and mental health research. Am. Psychol. 2003 Jan;58(1):64–74. doi: 10.1037/0003-066x.58.1.64. PMID: 12674819.	2003	1,129	3,208
2	Puchalski C, Ferrell B, Virani R, Otis-Green S, Baird P, Bull J, Chochinov H, Handzo G, Nelson-Becker H, Prince-Paul M, Pugliese K, Sulmasy D. Improving the quality of spiritual care as a dimension of palliative care: the report of the Consensus Conference. J Palliat Med. 2009 Oct;12(10):885–904. doi: 10.1089/jpm.2009.0142. PMID: 19807235.	2009	574	1,187
3	Anandarajah G, Hight E. Spirituality and medical practice: using the HOPE questions as a practical tool for spiritual assessment. Am Fam Physician. 2001 Jan 1;63(1):81–9. PMID: 11195773.	2001	288	870
4	Tanyi RA. Towards clarification of the meaning of spirituality. J Adv Nurs. 2002 Sep;39(5):500–9. doi: 10.1046/j.1365-2648.2002.02315.x. PMID: 12175360.	2002	274	845
5	Reed PG. Spirituality and well-being in terminally ill hospitalized adults. Res Nurs Health. 1987 Oct;10(5):335–44. doi: 10.1002/nur.4770100507. PMID: 3671781.	1987	236	718
6	Breitbart W. Spirituality and meaning in supportive care: spirituality- and meaning-centered group psychotherapy interventions in advanced cancer. Support Care Cancer. 2002 May;10(4):272–80. doi: 10.1007/s005200100289. Epub 2001 Aug 28. PMID: 12029426.	2002	220	561
7	Reed PG. An emerging paradigm for the investigation of spirituality in nursing. Res Nurs Health. 1992 Oct;15(5):349–57. doi: 10.1002/nur.4770150505. PMID: 1529119.	1992	198	614
8	Dyson J, Cobb M, Forman D. The meaning of spirituality: a literature review. J Adv Nurs. 1997 Dec;26(6):1183–8. PMID: 9429969.	1997	166	562
9	Chiu L, Emblen JD, Van Hofwegen L, Sawatzky R, Meyerhoff H. An integrative review of the concept of spirituality in the health sciences. West J Nurs Res. 2004 Jun;26(4):405–28. doi: 10.1177/0193945904263411. PMID: 15155026.	2004	142	370
10	Cook CC. Addiction and spirituality. Addiction. 2004 May;99(5):539–51. doi: 10.1111/j.1360-0443.2004.00715.x. Erratum in: Addiction. 2006 May;101(5):761. PMID: 15078228.	2004	118	399
11	McSherry W, Cash K. The language of spirituality: an emerging taxonomy. Int J Nurs Stud. 2004 Feb;41(2):151–61. doi: 10.1016/s0020-7,489(03)00114-7. PMID: 14725779.	2004	104	277
12	Martsolf DS, Mickley JR. The concept of spirituality in nursing theories: differing world-views and extent of focus. J Adv Nurs. 1998 Feb;27(2):294–303. doi: 10.1046/j.1365-2648.1998.00519.x. PMID: 9515639.	1998	102	331
13	Worthington EL Jr, Hook JN, Davis DE, McDaniel, M. A. Religion and spirituality. J Clin Psychol. 2011 Feb;67(2):204–14. doi: 10.1002/jclp.20760. PMID: 21108313.	2011	101	343
14	McSherry W, Cash K, Ross L. Meaning of spirituality: implications for nursing practice. J Clin Nurs. 2004 Nov;13(8):934–41. doi: 10.1111/j.1365-2702.2004.01006.x. PMID: 15533099.	2004	98	275
15	Newlin K, Knafl K, Melkus GD. African-American spirituality: a concept analysis. ANS Adv Nurs Sci. 2002 Dec;25(2):57–70. doi: 10.1097/00012272-200212000-00005. PMID: 12484641.	2002	93	291

**Table 2:** Most cited books, according to Google Scholar citations in November 3, 2020, found in the present study.

Ranking	Book	Year	No. Google Scholar citations
1	Koenig, H. G., McCullough, M., & Larson, D. B. (2001). Handbook of religion and health. New York: Oxford University Press.	2001	7,245
2	Koenig H. G., King D. & Carson V. (2012). Handbook of Religion and Health. Oxford University Press, New York.	2012	7,245
3	Stoll R. I. (1989). The essence of spirituality. ln: Carson V. B, ed. Spiritual Dimensions of Nursing Practice. Philadelphia: Saunders.	1989	388
4	Koenig, H. G. (2005). Faith and mental health: Religious resources for healing (p. 44). Philadelphia and London: Templeton Foundation Press.	2005	300
5	Solomon R. (2002). Spirituality for the skeptic: The thoughtful love of life. New York: Oxford Univ. Press.	2002	233
6	O’Brien, M. E. (1982). The need for spiritual integrity. In H. Yura & M. B. Walsh (Eds.), Human needs and the nursing process (pp. 85–115). Norwalk, CT: Appleton- Century-Crofts.	1982	66
7	Colliton, M. A. (1981). The spiritual dimension of nursing. In I. L. Beland & J. Y. Passes (eds.), Clinical Nursing (4th ed.) (pp. 492–501) New York, NY: Macmillan.	1981	61
8	Renetzky L. (1979) The fourth dimension: applications to the social services. In: Moberg D, ed. Spiritual Well Being. University Press of America, Washington: 215–28	1979	30
9	Walsh, R. (1999). Essential spirituality. The 7 Central Practices to Awaken Heart and Mind. New York: John Wiley	1999	22
10	Surbone, A., Konishi, T., & Baider, L. (2011). Spiritual issues in supportive cancer care. In I. N. Oliver (Ed.), The MASCC textbook of cancer supportive care (pp. 419–425). New York, NY: Springer	2011	3
11	Smeltzer, S., Bare, B., (1996). Brunner and Suddarth’s Textbook of Medical–Surgical Nursing. Lippincott Raven Publishers, Philadelphia, PA.	1996	3

With the above-mentioned procedures, 24 spirituality dimensions were found (Table 3). The dimensions were recognized from the identification of terms or expressions that were common to at least three different spirituality definitions, for example, terms such as “connection,” “God,” and “life after death.” A score was made to analyze how often a term appeared. All definitions used can be found in Supplementary Material.

**Table 3:** Spirituality dimensions.

Connection/Relation	53001%
Meaning/purpose	51.80%
Divine/god/higher power	39.75%
Transcendence/immaterial	38.55%
Others/community relationship	37.95%
Beliefs	29.51%
Self connection	25.90%
Nature connection	24.09%
Values	23.49%
Individual/personal	19.87%
Experience	19.87%
Practices/behaviors	18.67%
Peace/well-being	15.06%
Human aspect	13.85%
Power, force, inner energy	13.85%
Sacred	12.04%
Personal growth	10%
Immanence	5.42%
Support/sustain element	5.42%
Dynamic process	4.81%
Necessity	3.61%
Spiritual beings	3.01%
Art connection	1.80%
Life after death	1.80%

### Synthesized Findings

Most of the publications considered spirituality as a “connection” or “relation” (53.01%), which provides (or is the search for) purpose, meaning or reason for being (51.80%). Our results also found that the sense of spirituality connection occurs in relation to the Divine, God, or Higher Power (39.75%), in relation to something transcendent (38.55%), in relation to other people (37.95%), through self-connection (25.90%), and/or with nature (24.09%). In a less relevant way, there are connections with the sacred (12.04%), with an immanent aspect (5.42%), with spiritual/supernatural beings (3.01%), and through art (1.80%).

Three important dimensions were also found and can function as axes of spirituality: beliefs or faith (29.51%), experiences (19.87%), and practices or behaviors (18.67%). Furthermore, spirituality was presented as an intrinsically human characteristic (13.85%), as a subjective, individual, and particular aspect (19.87%), and as a dynamic process (4.81%). It could be felt as a power or inner energy (13.85%), as an element that sustains (5.42%), or it could be felt as a necessity to achieve (3.61%). It can also be understood as a life after death belief and attribute (1.80%). Finally, spirituality was related to the development of peace and well-being feelings (15.06%), values (23.49%), and personal growth (10%).

## Discussion

To our knowledge, this is the first study that systematically evaluated the most important and highly cited spirituality definitions under the healthcare field, instead of focusing on one specific area such as nursing or palliative care. We found a large body of scientific literature proposing and analyzing definitions of spirituality. If, on the one hand, this amount of articles shows the great interest concerning the association between spirituality aspects and healthcare, on the other hand, it shows that this is a controversial and challenging issue for the academic field, revealing a clear lack of consensus on the understanding of what spirituality is (George et al., 2000; Speck, 2005).

The most cited reference from an article included in our review comes from Hill and Pargament (2003), which states that “*spirituality can be understood as a search for the sacred, a process through which people seek to discover, hold on to, and, when necessary, transform whatever they hold sacred in their lives*” (Hill and Pargament, 2003). Among the books, the most cited reference comes from Koenig et al. (2001), “*Spirituality is the personal quest for understanding answers to ultimate questions about life, about meaning, and about relationship to the sacred or transcendent, which may (or may not) lead to or arise from the development of religious rituals and the formation of community*” (Koenig et al., 2001).

We discuss below the most important spirituality dimensions (“connection,” “interpretation of life,” “beliefs, practices and experiences,” “spirituality sensations”, and “spirituality as an intrinsic component of human beings”) found in these studies. Based on this theoretical background, we propose the organization of a framework that can be used for clinical practice, training of healthcare professionals and future research.

### Connection: Narrower or Broader

Connection (or relationship) could be considered as a central aspect of spirituality, as found in 88 definitions (53.01%). Broader definitions tend to consider spirituality as experiences of connection with nature, social relations, and art, while narrower definitions place spirituality in a more theistic approach (i.e., related to the Divine, God, a Higher Power, or the Transcendent). Analyzing this dimension, we can identify an overlapping of spiritual and religious meanings.

It can be observed, for example, in the definition of spirituality proposed by Bergamo and White (2016), “*(…) Most spiritual affiliations relate to surrendering personal control, searching for a larger life meaning, and recognizing a higher or transcendent power. Spirituality may also refer to more generalized feelings of connectedness with others or strong personal values that may assist individuals with finding peace and contentment in their lives. Spirituality is broad in definition as it may range from beliefs and connectedness to organized religion or may be based on more generalized personal values*” (Bergamo and White, 2016).

Historically, the understanding of spirituality was linked to the expression of religiosity. Religiosity can be described as the way an individual follows and experiences or practices a given religion, whether intrinsically or extrinsically, following an organizational and/or non-organizational standard (Allport and Ross, 1967; Koenig and Büssing, 2010). The term spirituality was used to designate the religious traditions of the East, upon the colonial encounters, and also it was apparently used within the Catholicism of the seventeenth century in a negative context, to describe subjective forms of religious practice (Wolfteich, 2005; Van Der Veer, 2008).

However, in the last decades, the distinction between spirituality and religiosity has been gaining more representativeness associated with the “new age” movement, which brought the approach of spirituality unrelated to religion, with the increase in the number of people who declare themselves atheist and “*spiritual but not religious*,” a group identified by Zinnbauer et al. (1997), Koenig (2008) and Zimmer et al. (2016) which can be understood as composed by individuals with a comprehensive spirituality connection, presenting a rather personal nature of spirituality (Marshall and Olson, 2018; Wixwat and Saucier, 2021).

Following this point of view, it is possible to note that for some authors, spirituality has been considered as something broader, which may involve religiosity, but goes beyond it (Jones et al., 2011). From our results, we recognize that spirituality and religiosity are related and overlapping, varying according to the cultural context and to the dynamic quality of the spirituality itself.

Understanding this relation may prevent a dualism in the understanding of spirituality, as something good, and religion, as something bad, noting instead that both can have positive and negative aspects in their expression (Hill et al., 2000). In addition, this overlapping supports that religious traditions should be understood by health professionals, particularly in clinical practice and in the training of health professionals (Puchalski and Larson, 1998).

### Interpretation of Life: Meaning and Purpose

Spirituality can be considered a source of coping to handle crisis and stressful moments, and related to positive meanings in face of challenges, such as in health problems. This process is related to improving patients’ outcomes, manages chronic pain, or deals with a diagnostic as cancer (Breitbart, 2002; Vachon, 2008; Dedeli and Kaptan, 2013; Weber and Pargament, 2014; Bernard et al., 2017).

For Reed (1992), based on the investigation of spirituality in nursing, “*Spirituality refers to the propensity to make meaning through a sense of relatedness to dimensions that transcend the self in such a way that empowers and does not devalue the individual*”(Reed, 1992). Other concepts, such as those from Koenig et al. (2001) and Puchalski et al. (2014), also highlight “meaning” as an important aspect of spirituality.

“Meaning” as a dimension of spirituality may exist in religious or non-religious individuals, pointing to an universal characteristic that can be used to assess the patient’s spirituality, as proposed in some assessment tools as the HOPE questions and FICA questionnaire (Anandarajah and Hight, 2001; Breitbart, 2002; Puchalski and Romer, 2005).

### Beliefs, Practices, and Experiences

Three important spirituality aspects were found in our search – beliefs, practices, and experiences – in agreement with the proposition of Anandarajah and Hight (2001), the third most cited article:

“*Spirituality is a complex and multidimensional part of the human experience. It has cognitive, experiential and behavior aspects. The cognitive or philosophic aspects include the search for meaning, purpose and truth in life and the beliefs and values by which an individual lives. The experiential and emotional aspects involve feelings of hope, love, connection, inner peace, comfort, and support. These are reflected in the quality of an individual’s inner resources, the ability to give and receive spiritual love and the types of relationships and connections that exist with self, the community, the environment and nature, and the transcendent* (e.g.*, power greater than self, a value system, God, cosmic consciousness*)*. The behavior aspects of spirituality involve the way a person externally manifests individual spiritual beliefs and inner spiritual state*” (Anandarajah and Hight, 2001).

Beliefs can be considered as the cognitive dimension of spirituality, an affirmation of something considered real, which varies according to the culture. Some religious/spiritual beliefs are, for example, the existence of a higher, transcendent power or the continuity of life after death. The belief of spirituality involving the existence beyond the death of the body could be found in studies with specific population, e.g., African–American women and Muslims (Banks-Wallace and Parks, 2004; Markani et al., 2013).

Practices correspond to the dimension of behavior, being social or individual, public or private, that requires the engagement of the individual to perform activities such as meditating, praying, or going to meetings of the group that shares his/her spiritual/religious beliefs.

The experiences compose the subjective aspect, based on the individual perception of the presence of elements of interaction with the connecting object of spirituality, going beyond the bond through the intellect.

These three components form a range that can be encouraged by the health professional when associated with health benefits, inviting the professional to a broader investigation on the relationship between health, spirituality, and the patient.

### Spirituality Sensations

Some spirituality definitions bring the description of feelings resulting from the spiritual connection experienced, such as in Tanyi (2002), one of the most cited articles:

“*(…) This connection brings faith, hope, peace, and empowerment. The results are joy, forgiveness of oneself and others, awareness and acceptance of hardship and mortality, a heightened sense of physical and emotional well-being, and the ability to transcend beyond the infirmities of existence*” (Tanyi, 2002).

Definitions based on concepts like peace, well-being, and quality of life tend to have a tautological problem, because these positive emotions cannot be distinguished from some measures of mental health (Koenig, 2008). On the other hand, it could be a way to identify whether negative spirituality exists, for example, if cases of “spiritual and religious problems” (DSM V - code 62.89) and “spiritual emergency” could be considered a negative aspect of spirituality because they are not associated with good feelings, values, and personal grow (Lukoff et al., 1998; Prusak, 2016).

### Spirituality as an Intrinsic Component of Human Beings

Puchalski et al. (2014) begin a spirituality definition from an international consensus as “*Spirituality is a dynamic and intrinsic aspect of humanity (…)*.” Understanding spirituality as a human characteristic could refer to ancient traditions of healthcare, such as Traditional Chinese Medicine and Ayurvedic Medicine (the traditional Indian medicine), which describe the human being as having also a spiritual component that was taken into account both to identify the cause of diseases and in their therapeutic approaches (Narayanasamy and Narayanasamy, 2006; Gureje et al., 2015; Mou, 2017; Teixeira, 2017). The approximation of conventional medicine to this point of view can be observed through the Integrative Medicine practices (Hu et al., 2015).

Even though spirituality can be understood as a characteristic common to all humans, it is a particular expression of each one. Individuality as a dimension of spirituality emphasizes the importance of looking at each person and their subjective experience, pointing to the person-centered care whose goal is a meaningful life, which is an important interception between health care and spirituality (Puchalski, 2013; Håkansson Eklund et al., 2019). The healthcare professionals should be capacitated in recognizing spiritual issues in their patients and facilitate connections with the appropriate support (Fitch and Bartlett, 2019).

Spirituality as an inner energy, a power or a force reminds us of the etymological origins of the word, its roots in the Latin “*spiritus*,” which roughly translates as “breath of life,” also “what animates,” “what gives life, existence.” A similar notion was carried by the terms “*ruah*,” in Hebrew, and “*pneuma*,” in Greek. It is interesting to note that these terms were traditionally used in a religious context for these societies (Cambridge International Dictionary of English, 1995; Longman Dictionary of Contemporary English, 2014; Withers et al., 2017).

## Proposed Framework for Spirituality in Healthcare

Based on the present systematic review, our findings allowed us to develop a framework for spirituality in healthcare, which will be discussed below.

The definitions of spirituality are multifactorial, including religious heritage, culture, generation, and nationality (Gall et al., 2011). Spirituality identified as a plural construct in a visual structure organization can guarantee an understanding of the complexity of this phenomenon. Based on learning theories, when content is exposed interconnecting the verbal and the visual, it facilitates the construction of connections, relationships, and understanding in the cognitive structure (Peer et al., 2021).

Spirituality can be analyzed through multiple dimensions, which identification may clarify how individuals interact with their spirituality, and which aspects greater impact more on health and treatment (Lunder et al., 2011; MacDonald et al., 2015). In this way, models of frameworks have been proposed in the literature, using relevant axes and constructs.

For example, McSherry et al. (2004) proposed a “Taxonomy of Spirituality” describing a spectrum of two ends: in one extreme, there is a spirituality based on religious and theist ideals, while at the other extreme there is a spirituality based upon secular, humanistic, and existential elements. A middle way is explained containing elements from both ends, but not as fundamental or radical. The elements are: theistic; religious affiliation; language; cultural, political and social ideologies; phenomenological; existential; quality of life and mystical. In another framework, Ko et al. (2017) propose that spirituality consists of two dimensions (i.e., vertical and horizontal) and eight attributes, considering which antecedents can lead to such dimension of spirituality and what are the consequences of it. In the horizontal dimension, the attributes are regarding connectedness with yourself or other people; in the vertical dimension, hierarchical, the connection is with God and about having a holy life (McSherry and Cash, 2004; Ko et al., 2017). Although there are some frameworks proposals on the concept of spirituality as seen above, there are still many aspects of this concept that remain unaddressed, indicating the need for a new, more comprehensive approach that may fit into the diverse cultural and religious contexts discussed in this research.

**Figure 2:**
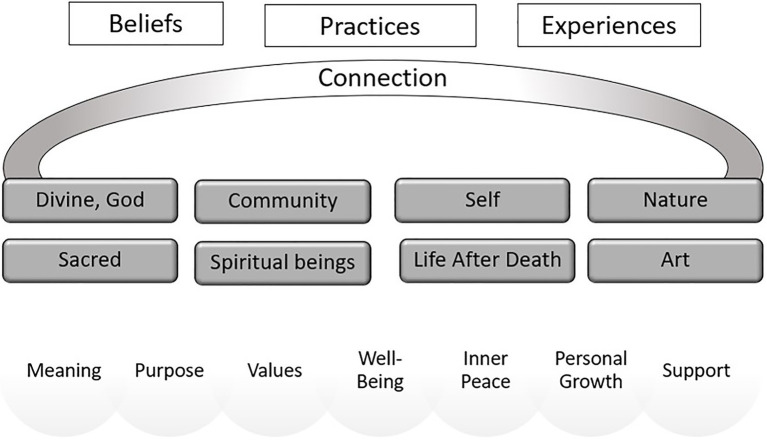
Spirituality framework proposition.

We present a spirituality framework proposition (Figure 2) that organizes all the dimensions found, except “human aspect,” “individual aspect,” “dynamic process,” and “necessity” because they can be understood as dimensions about the general spirituality nature, permeating all axes and cannot be dissociated and allocated to a single axis or dimension. The framework was designed as a didactic scheme, presenting the dimensions as a non-hierarchical and non-static construction, which flow according to individual’s context and experiences.

The representation of the dimensions of spirituality was divided into three axes/domains. The first axis (upper white section) is composed of beliefs, practices, and experiences that promote connection. It can be understood as a spirituality starter point. These aspects are assessed by some validated instruments as Spiritual Involvement and Beliefs Scale (SIBS) and Spiritual Well-Being Scale (SWBS; Hyman and Handal, 2006).

In the second axis (middle dark gray section) are the possible aspects that can be connected through spiritual beliefs, practices, or experiences. They are classified as:

- *Sacred* – something that cannot be described in ordinary, profane terms. Something can be considered sacred through a manifestation, a revelation to the individual or his/her religious/spiritual group, such as an object or symbol that reveals something of a unique nature to the person who contemplates it (Eliade, 2018).
- *Life after death* – related to the incorporeal, immaterial, and immortal portions present in the individual that survive in another realm after the body death. This beliefs in the immortality of the soul, in the existence of a spiritual dimension considering an extra-physical place, are found in some religions as Catholicism, Judaism, Hinduism and Buddhism (Siegel, 1980).
- *Spiritual beings* – related to the contact or influence of immaterial beings, even ancestors, that can connect to the material world through a paranormal sensitivity or anomalous experiences (Banks-Wallace and Parks, 2004; Martins and Zangari, 2012). Similar terms: Spirits, Ghosts, Supernatural presences.
- *Divine, God* – refers to the belief of one or more gods, beings of ultimate power connected to the celestial world, as a spirituality vertical dimension. Associated with religious context (Ko et al., 2017).
- *Self* – relates to the connection with oneself, the body, and the individual’s inner resources (Anandarajah and Hight, 2001).
- *Community* – aspects related to the ability to feel significative connection with other persons in the community, their neighbors, or family. This kind of connection could be understood as the social factor of the spirituality (Kao et al., 2020).
- *Nature* – understand the immanent nature as a mean of expression of the sacred. Already present in some aboriginal cultures, Celtic and Folk religions which respect all the nature as a living being. Also called “Ecospirituality” (Effa, 2017).
- *Art* – contemplate or develop an artwork (painting, sculpture, music, dance, literature, architecture) is an aesthetic experience that can stimulate the individual’s sensitive aspect leading him/her to the state of awe and/or to the perception of transcendence. The art can be seen in some spiritual cultures and religious rituals, for example, Buddhist sand mandalas and songs used in cults (Mooney and Timmins, 2007; Jones et al., 2019).

The third axis (lower gray section) refers to the development of values, personal growth, and sensations of meaning, purpose in life, well-being, support, and inner peace through connection with something that can affect the behavior of the individual. This perception is a concern for some religious faiths, to enhance this feeling that can be called “spiritual well-being” (Moberg, 1984).

We believe that the identification of these dimensions can help researchers and health professionals to map how individuals understand and express their spirituality, making it operational.

## Limitations

Several limitations can be identified in the present study. First, we only included one database and, for this reason, articles indexed only in other databases were not included; PubMed was chosen because this is a medical database and the definitions of spirituality included in these articles were more likely to be related to healthcare. Second, we used a narrow Boolean search that could impair a broader contextualization of the field. Although other terms such as “meaning” or “understanding” could have been used, they would result in a great number of unrelated references, since there are several articles assessing meaning of life or understanding the mechanisms for the relationship between R/S and health outcomes. Therefore, in order to focus our search on articles specifically providing a definition or concept, we chose to limit the terms used in the review. Third, there is a low multicultural representativeness because most of the articles were from the United States, in English, showing a possible bias of an Anglo-Saxon, Western, and Judeo-Christian culture, which may have impacted the definitions presented in the articles. Forth, although English is the main language in the scientific literature, the constructs of this framework need to be made for other languages that have large worldwide representation, since the contents described here may not apply to the linguistic variety in countries like India and China, for example. Further research is needed to explore the language issue in different population samples. Fifth, this framework is herein newly proposed, and thus, it still lacks a validation by other studies. Sixth, the credibility of the included references was assessed by authors reading the full text of each article, as well as assessing the usual scientometric parameters, i.e., number of citations, which could be considered another limitation. Particularly in this field of knowledge, theoretical and conceptual articles have limited space in high impact journals, and citations could be a good approach to see the impact of the definition/concept in the field. Another important point is that funders role on the manuscripts was not evaluated, posing risks of bias for the adopted definitions of each article. Seventh, we gave insufficient consideration to the manner in which the definitions of spirituality were developed (e.g., conceptual versus empirical approaches to definition and measurement) and it should be considered in further research.

## Conclusion

The tendency to expand the concept of health to embrace what is called spirituality gains strength with the academic evidence that relates health and spirituality. However, due to the lack of consensus on the term and the existing cultural gaps, we propose a new spirituality framework, based on a systematic review of the literature, in which spirituality (1) is a human individual, dynamic characteristic; (2) is expressed through beliefs, practices, and experiences in the search for connection with something that promotes meaning and personal growth; and (3) leads to the development of values and positive inner feelings.

Based on this, we aim to contribute to this field of knowledge, recognizing the areas of spirituality related to healthcare and the way in which it occurs, as well as to help in the classification and development of measurement instruments, thus creating an index for comparison of sample groups. The framework is intended to aid researchers in better characterizing what they mean by “spirituality,” so a clearer and less prone to interpretations use of the term can take place in the scientific literature. In this sense, what we herein propose is a common ground where elements of different components of spirituality, which are not usually associated, can be understood in a coherent scenario, helping researchers to better design and comprehend their findings, as well readers to build a common ground of knowledge.

## Data Availability Statement

The original contributions presented in the study are included in the article/Supplementary Material, further inquiries can be directed to the corresponding author.

## Author Contributions

MS and MP conceived of the presented idea. MS and RD collected the data and wrote the manuscript with support from GL and MP. GL aided in interpreting the results and edited the language. MP supervised the project. All authors contributed to the article and approved the submitted version.

## Conflict of Interest

The authors declare that the research was conducted in the absence of any commercial or financial relationships that could be construed as a potential conflict of interest.

The handling Editor declared a past co-authorship with GL and MP.

## Publisher’s Note

All claims expressed in this article are solely those of the authors and do not necessarily represent those of their affiliated organizations, or those of the publisher, the editors and the reviewers. Any product that may be evaluated in this article, or claim that may be made by its manufacturer, is not guaranteed or endorsed by the publisher.
