# Supplementary material for: Defining Spirituality in Healthcare: A Systematic Review and Conceptual Framework
Source: Front Psychol. 2021 Nov 18;12:756080. doi: 10.3389/fpsyg.2021.756080 (PMC8637184; doi:10.3389/fpsyg.2021.756080)
Supplement: Supplementary file 1 [file Table_1.DOCX]

**SUPPLEMENTARY MATERIAL**

| **REFERENCE** | **YEAR** | **DEFINITION** |
| --- | --- | --- |
| Anandarajah G, Hight E. Spirituality and medical practice: using the HOPE questions as a practical tool for spiritual assessment. Am Fam Physician. 2001 Jan 1;63(1):81-9. PMID: 11195773. | 2001 | Spirituality is a complex and multidimensional part of the human experience. It has cognitive, experiential and behavior aspects. The cognitive or philosophic aspects include the search for meaning, purpose and truth in life and the beliefs and values by which an individual lives. The experiential and emotional aspects involve feelings of hope, love, connection, inner peace, comfort and support. These are reflected in the quality of an individual’s inner resources, the ability to give and receive spiritual love and the types of relationships and connections that exist with self, the community, the environment and nature, and the transcendent (e.g., power greater than self, a value system, God, cosmic consciousness). The behavior aspects of spirituality involve the way a person externally manifests individual spiritual beliefs and inner spiritual state. |
| Anandarajah G. The 3 H and BMSEST models for spirituality in multicultural whole-person medicine. Ann Fam Med. 2008 Sep-Oct;6(5):448-58. doi: 10.1370/afm.864. PMID: 18779550; PMCID: PMC2532766. | 2008 | The 3 H’s encompass cognitive (head), experiential (heart), and behavioral (hands) aspects of the human spiritual experience. The phrase “head, heart, and hands”. is used widely in other contexts but is applicable here. The cognitive, or existential, (head) aspects include search for meaning and purpose, and values and beliefs most important in one’s life. The experiential (heart) aspects encompass the human need for love, inner peace, resilience, and connection. Finally, the behavioral (hands) aspects pertain to the outward expression of spiritual beliefs and needs, such as life choices, behavior toward others, rituals, and practices. These dimensions of spirituality are applicable to all human beings irrespective of culture or belief system, whether secular or religious |
| Appleby A, Swinton J, Bradbury I, Wilson P. GPs and spiritual care: signed up or souled out? A quantitative analysis of GP trainers' understanding and application of the concept of spirituality. Educ Prim Care. 2018 Nov;29(6):367-375. doi: 10.1080/14739879.2018.1531271. Epub 2018 Oct 19. PMID: 30339055. | 2018 | Very few GPs feel that spirituality is a meaningless concept, however many feel spirituality to be an unclear term. There is broad agreement that spirituality refers to a fundamental aspect of humanity, relating to a sense of personal meaning/purpose and is a psychological need. Most respondents feel the concept is useful and important to general practice. There were mixed views about whether spirituality implies a divine connection. |
| Appleby A, Swinton J, Wilson P. What GPs mean by 'spirituality' and how they apply this concept with patients: a qualitative study. BJGP Open. 2018 Apr 18;2(2):bjgpopen18X101469. doi: 10.3399/bjgpopen18X101469. PMID: 30564713; PMCID: PMC6184090. | 2018 | Spirituality is a meaningless concept, an unclear concept, useful concept, psychological need, personal meaning, integral to humanity, divine connection or relationship |
| Asgeirsdottir GH, Sigurbjörnsson E, Traustadottir R, Sigurdardottir V, Gunnarsdottir S, Kelly E. "To cherish each day as it comes": a qualitative study of spirituality among persons receiving palliative care. Support Care Cancer. 2013 May;21(5):1445-51. doi: 10.1007/s00520-012-1690-6. Epub 2013 Jan 4. PMID: 23288396. | 2013 | This included spirituality which was understood broadly as a vital element connected to seeking meaning, purpose and transcendence in life and touched the core of their existence. Spirituality was considered a part of everyday life and appeared in both religious and non-religious forms including the spiritual meaning of family relationships expressed as the chain of life providing the larger context of life, the meaning of God/a higher being and spiritual practices. Non-religious spirituality connected the participants to the larger context of family relations while religious spirituality was manifested through relationship with God/a higher being, as nine of the ten participants expressed faith. |
| Baker DC. Studies of the inner life: the impact of spirituality on quality of life. Qual Life Res. 2003;12 Suppl 1:51-7. doi: 10.1023/a:1023573421158. PMID: 12803311. | 2003 | DIMENSIONS: The first is that of the transcendent. The second dimension is that of a transpersonal viewpoint. we have the third dimension termed transmissional. |
| Banks-Wallace J, Parks L. It's all sacred: African American women's perspectives on spirituality. Issues Ment Health Nurs. 2004 Jan-Feb;25(1):25-45. doi: 10.1080/01612840490249028-22. PMID: 14660315. | 2004 | DIMENSIONS: Trusting God, Activities of daily living and mundane experiences were seen as opportunities for God to intervene in practical ways. Spirituality extended beyond death of the body, he continued role of the spirits of the ancestors within daily life. a belief in relationships continuing beyond death (continuing to communicate with and be nurtured by the deceased person) |
| Bash A. Spirituality: the emperor's new clothes? J Clin Nurs. 2004 Jan;13(1):11-6. doi: 10.1046/j.1365-2702.2003.00838.x. PMID: 14687288. | 2004 | Non theistic approach: This way of thinking is secular and has to do with values and meaning, about human development and endeavour./ Theistic approach: spirituality in some sort of transcendent force or being. / The via media: Spirituality is here described in secular terms but using language and thought forms to do with the transcendent. This approach is a muddle, for it engages with the transcendent but denies that the transcendent necessarily has anything to do with the question or the answer |
| Bergamo D, White D. Frequency of Faith and Spirituality Discussion in Health Care. J Relig Health. 2016 Apr;55(2):618-30. doi: 10.1007/s10943-015-0065-y. PMID: 25987303. | 2016 | Spirituality is multidimensional and has been described as an overarching way of life that informs thoughts and behaviors which may be personal or private in nature . Most spiritual affiliations relate to surrendering personal control, searching for a larger life meaning, and recognizing a higher or transcendent power . Spirituality may also refer to more generalized feelings of connectedness with others or strong personal values that may assist individuals with finding peace and contentment in their lives . Spirituality is broad in definition as it may range from beliefs and connectedness to organized religion or may be based on more generalized personal values. |
| Bienenfeld D, Yager J. Issues of spirituality and religion in psychotherapy supervision. Isr J Psychiatry Relat Sci. 2007;44(3):178-86. PMID: 18078252. | 2007 | Spirituality, at its broadest, is a person’s attempt to make sense of his/her world beyond the tangible and temporal. It strives to connect the individual with the transcendent and transpersonal elements of human existence. It may, but need not, include religion. |
| Borges Mda S, Santos MB, Pinheiro TG. Social representations about religion and spirituality. Rev Bras Enferm. 2015 Jul-Aug;68(4):524-31, 609-16. English, Portuguese. doi: 10.1590/0034-7167.2015680406i. PMID: 26422031. | 2015 | A espiritualidade como fé e crença em uma força superior |
| Boswell GE, Boswell-Ford KC. Testing a SEM model of two religious concepts and experiential spirituality. J Relig Health. 2010 Jun;49(2):200-11. doi: 10.1007/s10943-009-9254-x. Epub 2009 May 12. PMID: 19434496. | 2010 | only one dimension of spirituality (experiential spirituality) |
| Breitbart W. Spirituality and meaning in supportive care: spirituality- and meaning-centered group psychotherapy interventions in advanced cancer. Support Care Cancer. 2002 May;10(4):272-80. doi: 10.1007/s005200100289. Epub 2001 Aug 28. PMID: 12029426. | 2002 | DIMENSIONS: Meaning and Faith |
| Bruce A, Sheilds L, Molzahn A. Language and the (im)possibilities of articulating spirituality. J Holist Nurs. 2011 Mar;29(1):44-52. doi: 10.1177/0898010110381116. Epub 2010 Sep 14. PMID: 20841392. | 2011 | It is thought to encompass a state of peace and harmony and relates to ultimate questions about the meaning of life, illness, and death as well as belief in a divine being. |
| Bryson KA. Spirituality, meaning, and transcendence. Palliat Support Care. 2004 Sep;2(3):321-8. doi: 10.1017/s1478951504040428. PMID: 16594418. | 2004 | DIMENSIONS: 1 - spirituality is an innate tendency toward meaning, 2 - the spiritual finds expression in the temporal, 3 - though spirituality is animated by the transcendent, 4 - spirituality moves us toward the attainment of unity, truth, and goodness - compassion - , and 5 - spirituality is expressed in four areas of activity taking place: i) at the level of self, ii) other persons, iii) the natural environment, and iv) the unseen order |
| Buck HG. Spirituality: concept analysis and model development. Holist Nurs Pract. 2006 Nov-Dec;20(6):288-92. doi: 10.1097/00004650-200611000-00006. PMID: 17099417. | 2006 | spirituality is defined as: that most human of experiences that seeks to transcend self and find meaning and purpose through connection with others, nature, and/or a Supreme Being, which may or may not involve religious structures or traditions.DIMENSIONS: (1) intrinsically human—but not cognitively limited; (2) ontological and teleological; (3) self—transcendent; (4) connection with the corporeal and incorporeal (others, nature, and/or a Supreme Being); and (5) may or may not involve religious structures and traditions. |
| Burkhardt MA. Becoming and connecting: elements of spirituality for women. Holist Nurs Pract. 1994 Jul;8(4):12-21. doi: 10.1097/00004650-199407000-00004. PMID: 8027193. | 1994 | the unifying force that shapes and gives mean­ing to the pattem of one's self-becoming. This force is expressed in one' s being, in one's knowing, and in one's doing, and is experienced in caring connections with Self, Others, Nature, and God or Higher Power. |
| Canfield C, Taylor D, Nagy K, Strauser C, VanKerkhove K, Wills S, Sawicki P, Sorrell J. Critical Care Nurses' Perceived Need for Guidance in Addressing Spirituality in Critically Ill Patients. Am J Crit Care. 2016 May;25(3):206-11. doi: 10.4037/ajcc2016276. PMID: 27134224. | 2016 | That part of a person that gives meaning and purpose to the person’s life. Belief in a higher power that may inspire hope, seek resolution, and transcend physical and conscious constraints |
| Cervantes JM. Mestizo spirituality: toward an integrated approach to psychotherapy for Latina/OS. Psychotherapy (Chic). 2010 Dec;47(4):527-39. doi: 10.1037/a0022078. PMID: 21198240. | 2010 | Mestizo spirituality starts with the premise that traumas, emotional/physical insults, joys, and sufferings of life are part of one’s spiritual journey toward wholeness. Key concepts embedded in Mestizo spirituality and interwoven with the principles are noted in the following: 1 - Awareness, responsibility, respect, and kindness for the sacredness of one’s life journey./ 2 -Review and renewal of one’s religious/spiritual beliefs, traditions, and rituals./ 3 - forgiveness of one’s past wrong doings and reaffirmation of one’s connection to a larger cosmic reality. / 4 - Learning to become a person of knowledge/becoming impeccable, or ability to speak from one’s heart. /5 - Realization that service to others is the natural order of things. |
| Chao CS, Chen CH, Yen M. The essence of spirituality of terminally ill patients. J Nurs Res. 2002 Dec;10(4):237-45. doi: 10.1097/01.jnr.0000347604.89509.bf. PMID: 12522736. | 2002 | DIMENSIONS: The essence of spirituality of terminally ill patients - Communion with Nature (inspiration from the beauty of nature, creativity), Communion with Self (self-identity, wholeness, inner peace), Communion with Higher Being (faithfulness, hope, gratitude), Communion with Others (love, reconciliation) |
| Charzyńska E, Heszen-Celińska I. Spirituality and Mental Health Care in a Religiously Homogeneous Country: Definitions, Opinions, and Practices Among Polish Mental Health Professionals. J Relig Health. 2020 Feb;59(1):113-134. doi: 10.1007/s10943-019-00911-w. PMID: 31512031; PMCID: PMC6976552. | 2020 | DIMENSIONS: concerning the definitions of spirituality led to the identifcation of seven categories: (1) relationship, (2) transcendence, (3) dimension of functioning, (4) a specifc human characteristic, (5) searching for the meaning of life, (6) value-based lifestyle, and (7) elusiveness and indefnability. |
| Chaves LJ, Gil CA. Older people's concepts of spirituality, related to aging and quality of life. Cien Saude Colet. 2015 Dec;20(12):3641-52. English, Portuguese. doi: 10.1590/1413-812320152012.19062014. PMID: 26691790. | 2015 | DIMENSIONS: Support, Relation with the Sacred, and Transcendence |
| Chiu L, Emblen JD, Van Hofwegen L, Sawatzky R, Meyerhoff H. An integrative review of the concept of spirituality in the health sciences. West J Nurs Res. 2004 Jun;26(4):405-28. doi: 10.1177/0193945904263411. PMID: 15155026. | 2004 | Based on literature describe spirituality "as an individual experience , subjective experience, intrinsic experience , conscious experience, and moreover, a way of being . Spirituality is manifested through becoming (Burkhardt, 1993), grows in scope and power , and bears fruits of spirituality . Spirituality is a personal journey to discover meaning and purpose in life." |
| Clark M, Emerson A. Spirituality in Psychiatric Nursing: A Concept Analysis. J Am Psychiatr Nurses Assoc. 2020 Jan 30:1078390320902834. doi: 10.1177/1078390320902834. Epub ahead of print. PMID: 31999205. | 2020 | Based on a review of the psychiatric nursing literature, spirituality in psychiatric nursing occurred in the context of patients’ value-based thinking and capacity and willingness to interact with others. Spirituality named patients’ search for meaning and purpose and their striving to achieve connection with others or with the transcendent. Spirituality in psychiatric nursing led to consolation and both positive and negative coping. |
| Clyne B, O'Neill SM, Nuzum D, O'Neill M, Larkin J, Ryan M, Smith SM. Patients' spirituality perspectives at the end of life: a qualitative evidence synthesis. BMJ Support Palliat Care. 2019 Nov 26:bmjspcare-2019-002016. doi: 10.1136/bmjspcare-2019-002016. Epub ahead of print. PMID: 31771958. | 2019 | dimensions: spirituality was expressed in three broad subthemes: religious under standings, relationships, and existential or humanistic understandings. Spirituality was a source of support and comfort to help patients deal with their illness and current situations |
| Cobb M, Dowrick C, Lloyd-Williams M. Understanding spirituality: a synoptic view. BMJ Support Palliat Care. 2012 Dec;2(4):339-43. doi: 10.1136/bmjspcare-2012-000225. Epub 2012 Jul 31. PMID: 24654218. | 2012 | Spirituality, or the ways in which people relate to and seek an ultimate or sacred reality, is part of our mental, personal and social life: it is both experienced and expressed, it refers to both the tangible and the immaterial. spirituality is a feature and capacity of the system as a whole in which people express and experience spirituality individually, through others and through ‘objects’ that effect and mediate spirituality in the world. |
| Cohen HL, Thomas CL, Williamson C. Religion and Spirituality as Defined by Older Adults. J Gerontol Soc Work. 2008;51(3/4):284-299. doi: 10.1080/01634370802039585. PMID: 19042652. | 2008 | Caucasian Protestant group reflects the more universal definition of spirituality and the mystery and deeper connectedness of all things.For the Jewish group, spirituality provided a strong indication in the importance of observance of Jewish traditions and, particularly to those who had children, the performance of rituals. Also unique to the Jewish participants, spirituality was related to the importance of acceptance of other religions, including other branches of Judaism. |
| Cohen M. Introduction: Spirituality, quality of life, and nursing care. Qual Life 2:47-49, 1993 29. Hungelmann J, Kenkel-Rossi | 1993 | "',.. an affirmation of life in relationship with God, self, community, and environment that celebrates and nurtures wholeness," |
| Colliton, M. A. (1981). The spiritual dimension of nursing. In I. L. Beland & J. Y. Passes (eds.), Clinical Nursing (4th ed.) (pp. 492-501) New York, NY: Macmillan. | 1981 | the life principle that pervades a person’s entire being, including volitional, emotional, moral-ethical, intellectual, and physical dimensions, and generates a capacity for transcendent values ” (Colliton, 1981, P 492) |
| Contemporary issues in medicine--Communicatin in medicine: report III of the Medical School Objectives Project. Acad Med. 1999. Washington, DC: Association of American Medical Colleges. | 1999 | “found in all cultures and societies” and “is expressed in an individual’s search for ultimate meaning through participation in religion and/or belief in God, family, naturalism, rationalism, humanism, and the arts” |
| Cook CC. Addiction and spirituality. Addiction. 2004 May;99(5):539-51. doi: 10.1111/j.1360-0443.2004.00715.x. Erratum in: Addiction. 2006 May;101(5):761. PMID: 15078228. | 2004 | Spirituality is a distinctive, potentially creative and universal dimension of human experience arising both within the inner subjective awareness of individuals and within communities, social groups and traditions. It may be experienced as relationship with that which is intimately ‘inner’, immanent and personal, within the self and others, and/or as relationship with that which is wholly ‘other’, transcendent and beyond the self. It is experienced as being of fundamental or ultimate importance and is thus concerned with matters of meaning and purpose in life, truth and values. |
| Coyle J. Spirituality and health: towards a framework for exploring the relationship between spirituality and health. J Adv Nurs. 2002 Mar;37(6):589-97. doi: 10.1046/j.1365-2648.2002.02133.x. PMID: 11879423. | 2002 | as transcendence, meaning and purpose, connectedness, hope, and faith, work to produce health benefits in terms of prevention, recovery from illness, or coping with illness |
| Daaleman TP. Religion, spirituality, and the practice of medicine. J Am Board Fam Pract. 2004 Sep-Oct;17(5):370-6. doi: 10.3122/jabfm.17.5.370. PMID: 15355951. | 2004 | spirituality is a set of beliefs, stories, and practices that respond to the basic human desire to find life meaning and purpose that may or may not be linked to religious beliefs, practices, or communities. |
| David Berenson. A Systemic View of Spirituality: God and Twelve Step Programs as Resources in Family Therapy. Journal of Strategic and Systemic Therapies. 1990; Vol. 9, No. 1, pp. 59-70. https://doi.org/10.1521/jsst.1990.9.1.59 | 1990 | ‘‘Spirituality, as opposed to religion, connotes a direct, personal experience of the sacred unmediated by particular belief systems prescribed by dogma of by hierarchical structures of priests, ministers, rabbis, or guru." |
| Delgado C. A discussion of the concept of spirituality. Nurs Sci Q. 2005 Apr;18(2):157-62. doi: 10.1177/0894318405274828. PMID: 15802748. | 2005 | Spirituality is a way of perceiving reality in its entirety, hold-ing and realizing certain values and goals, and experiencing positive and satisfying behaviors and emotions in life. |
| Dobratz MC. Building a Middle-Range Theory of Adaptive Spirituality. Nurs Sci Q. 2016 Apr;29(2):146-53. doi: 10.1177/0894318416630090. PMID: 26980894. | 2016 | Adaptive spiritually is integrating one’s beliefs and values, religious practices, and cultural values in adapting to physical illness, loss, and life’s transitions |
| Dombeck M, Karl J. Spiritual issues in mental health care. J Relig Health. 1987 Sep;26(3):183-97. doi: 10.1007/BF01533119. PMID: 24302032. | 1987 | life principle that pervades and animates a person’s entire being, including emotional and volitional aspects of life.” (Dombeck & Karl, 1987, P 183) |
| Dyson J, Cobb M, Forman D. The meaning of spirituality: a literature review. J Adv Nurs. 1997 Dec;26(6):1183-8. PMID: 9429969. | 1997 | This framework primarily consists of the self, others and `God' and the relationship between them. Within this overall framework the emerging themes of meaning, hope, relatedness/connectedness, beliefs/belief systems and the expression of spirituality can be articulated. |
| Eckersley RM. Culture, spirituality, religion and health: looking at the big picture. Med J Aust. 2007 May 21;186(S10):S54-6. PMID: 17516885. | 2007 | "Spirituality is a deeply intuitive, but not always consciously expressed, sense of connectedness to the world in which we live. Its most common cultural representation is religion, an institutionalised system of belief and ritual worship that usually centres on a supernatural god or gods." |
| Egan M, Swedersky J. Spirituality as experienced by occupational therapists in practice. Am J Occup Ther. 2003 Sep-Oct;57(5):525-33. doi: 10.5014/ajot.57.5.525. PMID: 14527114. | 2003 | Essentially spirituality was defined as one’s beliefs about the world and one’s place in it and how one lives out these beliefs, through reflection and conscious actions. Especially important among these beliefs and actions were those regarding the interconnectedness of persons to themselves, to one another, and to God or some other intangible force beyond the self. |
| Elkins, D. N., Hedstrom, L. J., Hughes, L. L., Leaf, J. A., & Saunders, C. Toward a humanistic phenomenological spirituality: Definition, description, and measurement. Journal of Humanistic Psychology, 1988; 28, 5-18. doi:10.1177/0022167888284002 | 1988 | "spirituality is a way of being and experiencing that comes about through awareness of transcendent dimension and that is characterized by certain values in regard to self, others, nature, life and whatever one considers to be the ultimate" |
| Elkonin D, Brown O, Naicker S. Religion, spirituality and therapy: implications for training. J Relig Health. 2014 Feb;53(1):119-34. doi: 10.1007/s10943-012-9607-8. PMID: 22562170. | 2014 | spirituality as being an internal personal experience that differs from individual to individual. Spirituality is seen as dynamic and having the potential to develop as the individual develops. spirituality is seen as part of human nature and connectedness. spirituality was perceived as the internalization of religion, and spirituality was perceived as the expression of religion. spirituality was regarded as having more positive connotations |
| Emblen JD. Religion and spirituality defined according to current use in nursing literature. J Prof Nurs. 1992 Jan-Feb;8(1):41-7. doi: 10.1016/8755-7223(92)90116-g. PMID: 1573115. | 1992 | Personal life principle which animates transcendent quality of relationship with God or a god being." |
| Fowler DN, Rountree MA. Exploring the meaning and role of spirituality for women survivors of intimate partner abuse. J Pastoral Care Counsel. 2009 Fall-Winter;63(3-4):3-1-13. PMID: 20306932. | 2009 | DIMENSIONS: Affects Everything , Sustains, Inner Guidance, Fosters Resilience , Enhances Faith |
| Fradelos EC, Tzavella F, Koukia E, Papathanasiou IV, Alikari V, Stathoulis J, Panoutsopoulos G, Zyga S. Integrating chronic kidney disease patient's spirituality in their care: health benefits and research perspectives. Mater Sociomed. 2015 Oct;27(5):354-8. doi: 10.5455/msm.2015.27.354-358. Epub 2015 Oct 5. PMID: 26622206; PMCID: PMC4639341. | 2015 | Spirituality is a very debatable issue and the term has no single and widely agreed definition. The key components of spirituality were ‘meaning’, ‘hope’, ‘relatedness/connectedness’, and ‘beliefs/ beliefs systems’. Spirituality has been characterized as the quest for meaning in life, mainly through experiences and expressions of mind, in a unique and dynamic process different for each individua |
| Gaillard DS, Shaha M. La palce de la spiritualité dans les soins infirmiers: une revue de littérature [The role of spirituality in nursing care: a literature review]. Rech Soins Infirm. 2013 Dec;(115):19-35. French. PMID: 24490451. | 2013 | Spirituality can be defined as "a breath of life or as the central dimension of the human being that pervades every aspect of his life". It reflects the quest for meaning, value and relationship with oneself, others, and, for some, with God |
| Garssen B, de Jager Meezenbroek E. Response to Letter from P. Salander. Psycho-Oncology 2007;16:93–94. | 2007 | "On the basis of interviews with laymen and discussions among experts we have distinguished aspects such as experiencing meaning in life, inner peace, experiencing connectedness with nature, wonder, appreciation of life and experiencing a relationship with a higher being" |
| Gielen J, Bhatnagar S, Chaturvedi SK. Spirituality as an ethical challenge in Indian palliative care: A systematic review. Palliat Support Care. 2016 Oct;14(5):561-82. doi: 10.1017/S147895151500125X. Epub 2015 Oct 29. PMID: 26510891. | 2016 | DIMENSIONS spirituality in Indian PC patients through three dimensions: the relational dimension, the existential dimension, and the values dimension |
| Gijsberts MJ, Echteld MA, van der Steen JT, Muller MT, Otten RH, Ribbe MW, Deliens L. Spirituality at the end of life: conceptualization of measurable aspects-a systematic review. J Palliat Med. 2011 Jul;14(7):852-63. doi: 10.1089/jpm.2010.0356. Epub 2011 May 25. PMID: 21612502. | 2011 | DIMENSIONS three dimensions of our model: Spiritual Well-being (meaning and purpose, connectedness), Spiritual Cognitive Behavioral Context (relationships with others, beliefs), and Spiritual Coping (seek and express) |
| Goddard NC. 'Spirituality as integrative energy': a philosophical analysis as requisite precursor to holistic nursing practice. J Adv Nurs. 1995 Oct;22(4):808-15. doi: 10.1046/j.1365-2648.1995.22040808.x. PMID: 8708203. | 1995 | spirituality as integrative energy. |
| Gottheil EA, Groth-Marnat G. A grounded theory study of spirituality: using personal narratives suggested by spiritual images. J Relig Health. 2011 Jun;50(2):452-63. doi: 10.1007/s10943-010-9366-3. PMID: 20585857. | 2011 | Spirituality emerged as a human process motivated by suffering and the expectation of healing through transformation of the internal tate, through the connection with another, through seeking wisdom, and ultimately through the connection with a transcendent factor. This is a new dynamic dimension of the definition of spirituality absent in the extant definitions of spirituality. |
| Gould J, Wilson S, Grassau P. Reflecting on spirituality in the context of breast cancer diagnosis and treatment. Can Oncol Nurs J. 2008 Winter;18(1):34-46. English, French. doi: 10.5737/1181912x1813439. PMID: 18512567. | 2008 | Participants’ definitions of spirituality s included a reference to nature, a deity or higher power, a sense of personal growth, loving others, and interconnectedness. |
| Greenwald DF, Harder DW. The dimensions of spirituality. Psychol Rep. 2003 Jun;92(3 Pt 1):975-80. doi: 10.2466/pr0.2003.92.3.975. PMID: 12841474. | 2003 | dimensions: The four spiritual facrors were named Loving Connection to others, Self-effacing Alrmism, Blissful Transcendence, and Religiosity/Sacredness |
| Griffith J, Caron CD, Desrosiers J, Thibeault R. Defining spirituality and giving meaning to occupation: the perspective of community-dwelling older adults with autonomy loss. Can J Occup Ther. 2007 Apr;74(2):78-90. doi: 10.2182/cjot.06.0016. PMID: 17458367. | 2007 | Spirituality is defined in terms of its close links to religion and belief in a benevolent greater power |
| Hamilton JB. Religion and Spirituality in Healthcare: Distinguishing Related and Overlapping Concepts From an African American Perspective. Cancer Nurs. 2020 Jul/Aug;43(4):338-339. doi: 10.1097/NCC.0000000000000749. PMID: 32554981. | 2020 | Spirituality, on the other hand, has been conceptualized to occur on a more personal level and apart from affiliations with organized religious institutions. Conceptualizations of spirituality may include a search for answers to questions about life, a relationship to God, and making meaning of individual human experience through dimensions of connectedness: within oneself; to others and the environment; or, to God or other higher power |
| Heriot CS. Spirituality and aging. Holist Nurs Pract. 1992 Oct;7(1):22-31. doi: 10.1097/00004650-199210000-00007. PMID: 1447327. | 1992 | Spirituality is described as being concerned with the personal interpretation of life and the inner - resources of people. Heriot (1992) |
| Hermann CP. The degree to which spiritual needs of patients near the end of life are met. Oncol Nurs Forum 2007;34:70–8. | 2007 | spirituality was defined as the inherent quality of all humans that activates and drives the search for meaning and purpose in life. Spirituality involves all aspects of individuals as experienced in relationships with self, others, and a transcendent dimension. |
| Hill PC, Pargament KI. Advances in the conceptualization and measurement of religion and spirituality. Implications for physical and mental health research. Am Psychol. 2003 Jan;58(1):64-74. doi: 10.1037/0003-066x.58.1.64. PMID: 12674819. | 2003 | spirituality can be understood as a search for the sacred, a process through which people seek to discover, hold on to, and, when necessary, transform whatever they hold sacred in their lives (Pargament, 1997, 1999) |
| Ho RT, Chan CK, Lo PH, Wong PH, Chan CL, Leung PP, Chen EY. Understandings of spirituality and its role in illness recovery in persons with schizophrenia and mental-health professionals: a qualitative study. BMC Psychiatry. 2016 Apr 2;16:86. doi: 10.1186/s12888-016-0796-7. PMID: 27038910; PMCID: PMC4818963. | 2016 | Spirituality was commonly understood as an essential part of human beings as well as connectedness to others in the external world. |
| Honiball G, Geldenhuys D, Mayer CH. Acknowledging others as 'whole beings'. Managers' perceptions of spirituality and health in the South African workplace. Int Rev Psychiatry. 2014 Jun;26(3):289-301. doi: 10.3109/09540261.2014.881331. PMID: 24953148. | 2014 | Spirituality can be defined as a subjective experience of being connected with oneself, others and the entire universe |
| Hurlbut J, Ditmyer M. Defining the Meaning of Spirituality Through a Qualitative Case Study of Sheltered Homeless Women. Nurs Womens Health. 2016 Feb-Mar;20(1):52-62. doi: 10.1016/j.nwh.2015.12.004. Epub 2016 Feb 12. PMID: 26902440. | 2016 | DIMENSIONS: five themes: (a) Belief in God or a Higher Power; (b) Distinction Between Religion and Spirituality; (c) Belief That There Is a Plan for Their Lives; (d) Spirituality Providing Guidance for What Is Right/Wrong; and (e) Belief That Their Lives Will Improve |
| Hyman, C., & Handal, P. J. Definitions and evaluation of religion and spirituality items by religious professionals: A pilot study. Journal of Religion and Health. 2006. 45(2), 264-282. | 2006 | "spirituality was define as subjective, internal and as either a divine experience or direct relationship with God./ Spirituality, however, is one’s search for the sacred that can be viewed subjectively, occurs internally, and is pursued through seeking a relationship with whatever one holds sacred." |
| Janse van Rensburg AB, Poggenpoel M, Myburgh CP, Szabo CP. Defining and Measuring Spirituality in South African Specialist Psychiatry. J Relig Health. 2015 Oct;54(5):1839-55. doi: 10.1007/s10943-014-9943-y. PMID: 25266141. | 2015 | The attributes of this definition included that spirituality constitutes a "quality", a "journey", a "relationship" as well as a "capacity". |
| Janse van Rensburg BA, Poggenpoel M, Myburgh CP, Szabo CP. A model for the role of defined spirituality in South African specialist psychiatric practice and training. J Relig Health. 2014 Apr;53(2):393-412. doi: 10.1007/s10943-012-9644-3. PMID: 23099614. | 2014 | the operational definition of spirituality for the purposes of this study is In individual persons and societies, the progressive inner: – quality of transcendental awareness; – journey towards understanding of ultimate questions; – relationship or connectedness (with themselves, others, the natural world and a theist or atheist presence/source/principle beyond themselves); and – capacity or consciousness concerning an unseen but vital, animating, life defining principle, force or energy within;  through which meaning and purpose are derived. |
| Johnson R, Hauser J, Emanuel L. Toward a clinical model for patient spiritual journeys in supportive and palliative care: Testing a concept of human spirituality and associated recursive states. Palliat Support Care. 2020 Jul 30:1-6. doi: 10.1017/S1478951520000607. Epub ahead of print. PMID: 32729457. | 2020 | spirituality defined as a construct that “involves concepts of ‘faith’ where faith is a belief in a higher transcendent power, but not necessarily identified as God (...)spirituality can be experienced as something in and of itself or as part of the physical, social, and psychological domains. |
| Jones S, Sutton K, Isaacs A. Concepts, Practices and Advantages of Spirituality Among People with a Chronic Mental Illness in Melbourne. J Relig Health. 2019 Feb;58(1):343-355. doi: 10.1007/s10943-018-0673-4. PMID: 30056485. | 2019 | Spirituality as part of a religion (Prctices include Prayer, reading in scripture, being in nature, meditation, yoga), Spirituality outside religion ( in nature, in art, New age spirituality - type of spirituality that beliefs usually encompass the entire universe within which humans are a part ). |
| Khantzian EJ, Mack JE. How AA works and why it's important for clinicians to understand. J Subst Abuse Treat. 1994 Mar-Apr;11(2):77-92. doi: 10.1016/0740-5472(94)90021-3. PMID: 8040921. | 1994 | Spirituality as defined by Khantzian and Mack (1994, pp. 83 and 90) is characterized as follows: “It usually refers to a deep sense that there exists in the universe a deeper structure of being, a purpose or possibility, or even a divine design. This divine design may be experienced as mysterious, or not readily manifest, but it can be approached or experienced through surrendering one’s egoistic sense of separateness and self-focused strivings” |
| Khorami Markani A, Yaghmaei F, Khodayari Fard M. Spirituality as experienced by Muslim oncology nurses in Iran. Br J Nurs. 2013 Feb 28-Mar 13;22(4):S22-4, S26-8. doi: 10.12968/bjon.2013.22.sup2.s22. PMID: 23448951. | 2013 | DIMENSIONS: The themes identified by the participants’ understanding of spirituality were searching for God, life mission and purpose, belief in life after death, improving communication, and transcendence. |
| King MB, Koenig HG. Conceptualising spirituality for medical research and health service provision. BMC Health Serv Res. 2009 Jul 13;9:116. doi: 10.1186/1472-6963-9-116. PMID: 19594903; PMCID: PMC2722588. | 2009 | Spirituality is the personal quest for understanding answers to ultimate questions about life, about meaning and about relationship to the sacred or transcendent, which may (or may not) lead to or arise from the development of religious rituals and the formation of community |
| Klingemann H, Schläfli K, Steiner M. "What do you mean by spirituality? Please draw me a picture!" Complementary faith-based addiction treatment in Switzerland from the client's perspective. Subst Use Misuse. 2013 Sep;48(12):1187-202. doi: 10.3109/10826084.2013.803875. PMID: 24041181. | 2013 | Important dimension: Connection with nature |
| Ko IS, Choi SY, Kim JS. [Evolutionary Concept Analysis of Spirituality]. J Korean Acad Nurs. 2017 Apr;47(2):242-256. Korean. doi: 10.4040/jkan.2017.47.2.242. Erratum in: J Korean Acad Nurs. 2017 Oct;47(5):712. PMID: 28470161. | 2017 | Spirituality was found to consist of two dimensions and eight attributes: 1)vertical dimension: ‘intimacy and connectedness with God’ and ‘holy life and belief’, 2) horizontal dimension: ‘self-transcendence’, ‘meaning and purpose in life’, ‘self-integration’, and ‘self-creativity’ in relationship with self, ‘connectedness’ and ‘trust’ in relationship with others·neighbors·nature. |
| Koenig H.G., King D. & Carson V. (2012) Handbook of Religion and Health. Oxford University Press, New York. | 2012 | Spirituality is distinguished from other things – humanism, values, morals and mental health – by its connection to the transcendent. The transcendent is that which is outside of the self, and yet also within the self – and in Western traditions is called God, Allah, HaShem, or a Higher Power and in Eastern traditions is called Ultimate Truth or Reality, Vishnu, Krishna, or Buddha. Spirituality is intimately connected to the supernatural and religion, although also extends beyond religion (and begins before it). Spirituality includes a search for the transcendent and so involves traveling along the path that leads from non-consideration to a decision not to believe to questioning to belief to devotion to surrender (Koenig et al. 2012, p. 46). |
| Koenig, H. G. (2005c). Faith and mental health: Religious resources for healing (p. 44). Philadelphia and London: Templeton Foundation Press. | 2005 | Spirituality involves a more generic personal quest for understanding answers to ultimate questions about life and its meaning, and while concerned with a relationship to the sacred or transcendent, may or may not lead to religious beliefs, rituals, or the formation of a community….. It may even be entirely divorced from religion (Koenig 2005). |
| Koenig, H. G., McCullough, M., & Larson, D. B. (2000). Handbook of religion and health. New York: Oxford University Press. | 2000 | “Spirituality is the personal quest for understanding answers to ultimate questions about life, about meaning, and about relationship to the sacred or transcendent, which may (or may not) lead to or arise from the development of religious rituals and the formation of community” (Koenig et al., 2000, p. 18) |
| Labun E. Spiritual care: an element in nursing care planning. J Adv Nurs. 1988 May;13(3):314-20. doi: 10.1111/j.1365-2648.1988.tb01424.x. PMID: 3417925. | 1988 | is an aspect of the total person that influences as well as acts in conjunction with other aspects of the person is related to and integrated with the functioning and expression of all other aspects of the person; has a relational nature which is expressed through interpersonal relationships between persons and through a transcendent relationship with another realm; involves relationships and produces behaviors and feelings which demonstrate the existence of love, faith, hope and trust, therein providing meaning to life and a reason for being.” (Labun, 1988, pp. 314-315) |
| Laukhuf G, Werner H. Spirituality: the missing link. J Neurosci Nurs. 1998 Feb;30(1):60-7. doi: 10.1097/01376517-199802000-00007. PMID: 9604824. | 1998 | "It is a personal, individual value system about the way people approach life. (...) Spirituality involves a personal quest to find meaning and purpose in life and relashionship to the miystery/God and the rest of universe." |
| Lauver DR. Commonalities in women's spirituality and women's health. ANS Adv Nurs Sci. 2000 Mar;22(3):76-88. doi: 10.1097/00012272-200003000-00007. PMID: 10711806. | 2000 | women's spirituality is the immanence of the Divine, is the ability to be self-affirming and other-affirming for life-enhancing mutuality, honors the fundamental relationships among all life forms, |
| Lavorato Neto G, Rodrigues L, Silva DARD, Turato ER, Campos CJG. Spirituality review on mental health and psychiatric nursing. Rev Bras Enferm. 2018;71(suppl 5):2323-2333. doi: 10.1590/0034-7167-2016-0429. PMID: 30365801. | 2018 | Spirituality is a question of meaning, a human symbolic construction on the purpose of life that covers the whole existence in a holistic perspective; it works through a transcendental sense of connectedness that bridges the sacred and common worlds. In a sacred way it expresses a connection with high powers, and deities), and religion may participate here. Connection with the common world means interlinking with others and circumstances. |
| Lepherd L. Spirituality in men with advanced prostate cancer: "it's a holistic thing . . . it's a package". J Holist Nurs. 2014 Jun;32(2):89-101; quiz 102-3. doi: 10.1177/0898010113504492. Epub 2013 Sep 30. PMID: 24080341. | 2014 | holistic spirituality: the centrality of connectedness with the associated aspects of process and journey, purpose and meaning, values, and peace and fulfillment. |
| Lepherd L. Spirituality: Everyone has it, but what is it? Int J Nurs Pract. 2015 Oct;21(5):566-74. doi: 10.1111/ijn.12285. Epub 2014 Mar 26. PMID: 24666816. | 2015 | Spirituality - is based on transcendence - as a state of the human spirit; religiousness; sacredness. Internal dimensions: Integrative energy, force; Values (including love, beliefs, faith); Process: (journey); Connectedness (self, others, higher being, other place); Existencial (purpose, meaning in life)/ Manifestation through behaviours (Internal or Internal and External): Meditation, reflection, mindfulness. Concept and practice of values: love, forgiveness, hope. Religion; ritual prayer, pilgrimage, community. / oUTCOMES iNTERNAL: Peace of mind; harmony; confort; alleviation of suffering; self-fulfilment; being, knowing and doing . |
| Lewis LM, Hankin S, Reynolds D, Ogedegbe G. African American spirituality: a process of honoring God, others, and self. J Holist Nurs. 2007 Mar;25(1):16-23; discussion 24-5. doi: 10.1177/0898010106289857. PMID: 17325309. | 2007 | DIMENSIONS There were three core categories of spirituality: love in action, relationships and connections, and unconditional love. |
| MacGillivray PS, Sumsion T, Wicks-Nicholls J. Critical elements of spirituality as identified by adolescent mental health clients. Can J Occup Ther. 2006 Dec;73(5):295-302. doi: 10.2182/cjot.06.006. PMID: 17201102. | 2006 | DIMENSIONS: The items rated highest (and thus were considered most relevant to spirituality) were those items that were related to an intangible part of the self (e.g. one's soul). the items were arranged into categories the top five categories: "an intangible part of the self ", "knowing yourself and the process of getting to know yourself better", "one's beliefs and values", "one's dreams and their pursuit", and "the search for the meaning/purpose of life". |
| Magura S, Knight EL, Vogel HS, Mahmood D, Laudet AB, Rosenblum A. Mediators of effectiveness in dual-focus self-help groups. Am J Drug Alcohol Abuse. 2003 May;29(2):301-22. doi: 10.1081/ada-120020514. PMID: 12765208; PMCID: PMC1828912. | 2003 | “a personal orientation that every person is allowed to define for him or herself, not a religious practice” (p. 306) |
| MahdiNejad JE, Azemati H, Sadeghi Habibabad A. Religion and Spirituality: Mental Health Arbitrage in the Body of Mosques Architecture. J Relig Health. 2020 Jun;59(3):1635-1651. doi: 10.1007/s10943-019-00949-w. PMID: 31722054. | 2020 | the spirituality is “a transcendental relationship and creation of a unity between the nature and human, and achievement of oneness and union with the universe” or the relationship between the individual and God, entrusting the affairs to him, trusting his all-out power and guidance |
| Mahlungulu SN, Uys LR. Spirituality in nursing: an analysis of the concept. Curationis. 2004 May;27(2):15-26. doi: 10.4102/curationis.v27i2.966. PMID: 15974016. | 2004 | spirituality was defined as an individual quest for a transcendent relationship by establishing and or maintaining a dynamic relationship with God / supernatural being as understood by the person and with significant others |
| Martsolf DS, Mickley JR. The concept of spirituality in nursing theories: differing world-views and extent of focus. J Adv Nurs. 1998 Feb;27(2):294-303. doi: 10.1046/j.1365-2648.1998.00519.x. PMID: 9515639. | 1998 | dimensions: Meaning, Value, Transcendence , Connecting ,Becoming |
| McCormick DP, Holder B, Wetsel MA, Cawthon TW. Spirituality and HIV disease: an integrated perspective. J Assoc Nurses AIDS Care. 2001 May-Jun;12(3):58-65. doi: 10.1016/s1055-3290(06)60144-1. PMID: 11387805. | 2001 | Spirituality is an intrinsic energy source that has a basis in both religion and existentialism. all individuals have needs for meaning in life, hope, and self-transcendence and that these needs are met by utilizing aspects of both religion and existentialism |
| McDowell D, Galanter M, Goldfarb L, Lifshutz H. Spirituality and the treatment of the dually diagnosed: an investigation of patient and staff attitudes. J Addict Dis. 1996;15(2):55-68. doi: 10.1300/J069v15n02_05. PMID: 8704001. | 1996 | Spirituality can refer to people who are concerned with metaphysical issues as well as their day to day lives. It need not connote God. |
| McNeil SB. Spirituality in Adolescents and Young Adults With Cancer: A Review of Literature. J Pediatr Oncol Nurs. 2016 Jan-Feb;33(1):55-63. doi: 10.1177/1043454214564397. Epub 2015 Jan 30. PMID: 25637188. | 2016 | DIMENSIONS: The elements of spirituality most commonly identified in these analyses include the following: transcendence, a search for meaning or purpose in life, a connection with others, and a personal belief system |
| McSherry W, Cash K, Ross L. Meaning of spirituality: implications for nursing practice. J Clin Nurs. 2004 Nov;13(8):934-41. doi: 10.1111/j.1365-2702.2004.01006.x. PMID: 15533099. | 2004 | DIMENSIONS: spirituality was about their essence, about what makes them unique, individual and ‘whole. /as a force that permeated every aspect of their life and being/.spirituality is a universal concept. It applies to the religious and non-religious./ the concept of spirituality within the context of supernatural, or spiritualist forces. |
| McSherry W, Cash K. The language of spirituality: an emerging taxonomy. Int J Nurs Stud. 2004 Feb;41(2):151-61. doi: 10.1016/s0020-7489(03)00114-7. PMID: 14725779. | 2004 | Taxonomy - Definition Range: Theistic, Religious, Language, Cultural, political, social ideologies; Phenomenological, Existential, Quality of life, Mystical. The taxonomy implies that an individual's worldview will determine their definition of spirituality. |
| McSherry W, Jamieson S. The qualitative findings from an online survey investigating nurses' perceptions of spirituality and spiritual care. J Clin Nurs. 2013 Nov;22(21-22):3170-82. doi: 10.1111/jocn.12411. PMID: 24118520. | 2013 | dimensions: Essence of the individual (individual perceptions), Essence of what makes them an unique human being (Personal values, System of beliefs, True self, Unique), My spirit is the inner most part of my being(Soul, Seat of emotions Physical, Core of Being), On our inner feelings, A sense of inner peace and acceptance (Balancing, Peace and acceptance, Applicable to all religion, Strong belief) |
| Mehnert, A. Sinnfindung und Spiritualität bei Patienten mit chronischen körperlichen Erkrankungen. Bundesgesundheitsbl. 49, 780–787 (2006). https://doi.org/10.1007/s00103-006-0008-6 | 2006 | the belief in a higher one transcendent power, to the divine and the meaningfulness of being |
| Melhem GA, Zeilani RS, Zaqqout OA, Aljwad AI, Shawagfeh MQ, Al-Rahim MA. Nurses' Perceptions of Spirituality and Spiritual Care Giving: A Comparison Study Among All Health Care Sectors in Jordan. Indian J Palliat Care. 2016 Jan-Mar;22(1):42-9. doi: 10.4103/0973-1075.173949. PMID: 26962280; PMCID: PMC4768449. | 2016 | "Spirituality is an abstract, subjective, and complex term, whose definition varies between individuals, philosophies, and cultures, and which has abstract components associated with many subjective meanings. In Muslim perspectives, spirituality is viewed as inseparable from their religion and as being derived from the Holy Qur'an and the Hadiths " |
| Memaryan N, Rassouli M, Mehrabi M. Spirituality Concept by Health Professionals in Iran: A Qualitative Study. Evid Based Complement Alternat Med. 2016;2016:8913870. doi: 10.1155/2016/8913870. Epub 2016 Jul 17. PMID: 27493675; PMCID: PMC4967431. | 2016 | “Spirituality is the sublime aspect of human existence bestowed on all humans in order for them to traverse the path of transcendence that is closeness to God (Allah)." |
| Meraviglia MG. Critical analysis of spirituality and its empirical indicators. Prayer and meaning in life. J Holist Nurs. 1999 Mar;17(1):18-33. doi: 10.1177/089801019901700103. PMID: 10373840. | 1999 | Spirituality is defined as experiences and expressions of one’s spirit in a unique and dynamic process reflecting faith in God or a supreme being; it is connectedness with oneself, others, nature, or God; and an integration of the dimensions of mind, body, and spirit |
| Meraviglia MG. The effects of spirituality on well-being of people with lung cancer. Oncol Nurs Forum. 2004 Jan-Feb;31(1):89-94. doi: 10.1188/04.ONF.89-94. PMID: 14722592. | 2004 | Spirituality was defined as the experiences and expressions of a person’s spirit in a unique and dynamic process reflecting faith in God or a supreme being; connectedness with self, others, nature, or God; and Integration of the dimensions of mind, body and spirit |
| Mesquita AC, Caldeira S, Chaves E, Carvalho EC. An Analytical Overview of Spirituality in NANDA-I Taxonomies. Int J Nurs Knowl. 2018 Jul;29(3):200-205. doi: 10.1111/2047-3095.12172. Epub 2017 Mar 1. PMID: 28247596. | 2018 | DIMENSIONS: Meaning in life, Connection, Transcendence, Values and Beliefs, Comfort, Well-Being, Life Principles |
| Misiorek A, Janus E. Spirituality in Occupational Therapy Practice According to New Graduates. OTJR (Thorofare N J). 2019 Oct;39(4):197-203. doi: 10.1177/1539449218808278. Epub 2018 Oct 31. PMID: 30379122. | 2019 | In the present study, the majority of persons associated spirituality with inner thoughts, human nature, and as the driving force controlling human choices; only a few persons stated that spirituality is connected with a higher power and is not connected directly with a particular person. |
| Moberg, D. O. Spirituality and aging: Research and implications. Journal of Religion, Spirituality, & Aging. 2008. 20, 95-134. doi:10.1080/15528030801922038 | 2008 | spirituality typically has a more existential and experiential focus upon an individual’s internalized faith, values, and beliefs along with their consequences in daily behavior. |
| Mok E, Wong F, Wong D. The meaning of spirituality and spiritual care among the Hong Kong Chinese terminally ill. J Adv Nurs. 2010 Feb;66(2):360-70. doi: 10.1111/j.1365-2648.2009.05193.x. PMID: 20423419. | 2010 | DIMENSIONS: Life is an integrated whole (Integration of mind and spirit, A unique personal belief and experience), Acceptance of death as a life process (Harmony with self and nature, Letting go), Finding meaning in life (Receiving and giving love in relationships and connectedness, Having faith in God/higher power, Being a good person), Having a sense of peace |
| Muldoon M, King N. Spirituality, health care, and bioethics. J Relig Health. 1995 Winter;34(4):329-49. doi: 10.1007/BF02248742. PMID: 11660133. | 1995 | “the way in which people understand and live their lives in view of their ultimate meaning and value” |
| Murgia C, Notarnicola I, Rocco G, Stievano A. Spirituality in nursing: A concept analysis. Nurs Ethics. 2020 Aug;27(5):1327-1343. doi: 10.1177/0969733020909534. Epub 2020 Apr 13. PMID: 32281485. | 2020 | Being part of a whole or a greater being is the entirety and the balance between body, mind and spirit in relation to and in total harmony with oneself, with others, with God and with nature. This is a transcendental expression and a dimension of spirituality |
| Nagai-Jacobson MG, Burkhardt MA. Spirituality: cornerstone of holistic nursing practice. Holist Nurs Pract. 1989 May;3(3):18-26. doi: 10.1097/00004650-198905000-00006. PMID: 2768352. | 1989 | . . . The essence of one's being,., the integrating or unifying factor, that which gives meaning and purpose |
| Nahardani SZ, Ahmadi F, Bigdeli S, Soltani Arabshahi K. Spirituality in medical education: a concept analysis. Med Health Care Philos. 2019 Jun;22(2):179-189. doi: 10.1007/s11019-018-9867-5. PMID: 30206758. | 2019 | It is the realization of an elevated meaning for life in relation to all aspects of one’s existence, including a wide spectrum of religiosity. |
| Narayanasamy A, Gates B, Swinton J. Spirituality and learning disabilities: a qualitative study. Br J Nurs. 2002 Jul 25-Aug 7;11(14):948-57. doi: 10.12968/bjon.2002.11.14.10467. PMID: 12165726. | 2002 | spirituality refers to that aspect of human existence that gives it its ‘humanness’. It concerns the structures of significance which give meaning and direction to a person’s life and helps him/her deal with the vicissitudes of existence. As such it includes vital dimensions such as the quest for meaning, purpose, self-transcending knowledge, meaningful relationships, love and commitment, as well as the sense of the Holy amongst us. A person’s spirituality is that part of them which drives them on |
| Narayanasamy A. A review of spirituality as applied to nursing. Int J Nurs Stud. 1999 Apr;36(2):117-25. doi: 10.1016/s0020-7489(99)00007-3. PMID: 10376221. | 1999 | Spirituality is rooted in an awareness which is part of the biological make up of the human species. Spirituality is present in all individuals, and it may manifest as inner peace and strength derived from perceived relationship with a transcendent God |
| Narayanasamy A. The puzzle of spirituality for nursing: a guide to practical assessment. Br J Nurs. 2004 Oct 28-Nov 10;13(19):1140-4. doi: 10.12968/bjon.2004.13.19.16322. PMID: 15573007. | 2004 | Spirituality: ‘‘Gives us a sense of personhood and individuality. It is the guiding force behind our uniqueness and acts as an inner source of power and energy, which makes us ‘tick over’ as a person. Spirituality is the inner, intangible dimension that motivates us to be connected with others and our surrounding. It drives us to search for meaning and purpose and establish positive and trusting relationships with others’’. |
| Newlin K, Knafl K, Melkus GD. African-American spirituality: a concept analysis. ANS Adv Nurs Sci. 2002 Dec;25(2):57-70. doi: 10.1097/00012272-200212000-00005. PMID: 12484641. | 2002 | DIMENSIONS: African-American spirituality envolves quintessential, internal, external, consoling, and transformative attributive dimensions |
| Niu Y, McSherry W, Partridge M. An understanding of spirituality and spiritual care among people from Chinese backgrounds: A grounded theory study. J Adv Nurs. 2020 Aug 4. doi: 10.1111/jan.14474. Epub ahead of print. PMID: 32748978. | 2020 | DIMENSIONS: understanding of spirituality and spiritual care, using three categories ‘essence and nature of life’,‘driving forces’, and ‘self-support’ |
| Noble A, Jones C. Getting it right: oncology nurses' understanding of spirituality. Int J Palliat Nurs. 2010 Nov;16(11):565-9. doi: 10.12968/ijpn.2010.16.11.80022. PMID: 21135791. | 2010 | it as being individual to each person and related to wellbeing. |
| Nolan MT, Mock V. A conceptual framework for end-of-life care: a reconsideration of factors influencing the integrity of the human person. J Prof Nurs. 2004 Nov-Dec;20(6):351-60. doi: 10.1016/j.profnurs.2004.08.007. PMID: 15599868. | 2004 | Spirituality as encompassing belief in or experience of the transcendent. |
| O’Brien, M. E. (1982). The need for spiritual integrity. In H. Yura & M. B. Walsh (Eds.), Human needs and the nursing process (pp. 85-115). Norwalk, CT: Appleton- Century-Crofts. | 1982 | is that which inspires in one the desire to transcend the realm of the material. |
| O'Connor TS, Meakes E, McCarroll-Butler P, Gadowsky S, O'Neill K. Making the most and making sense: ethnographic research on spirituality in palliative care. J Pastoral Care. 1997 Spring;51(1):25-36. doi: 10.1177/002234099705100104. PMID: 10169309. | 1997 | Spirituality is meaning making, making the most of life now, and making sense (meaning) of life |
| Ohajunwa C, Mji G. The African Indigenous Lens of Understanding Spirituality: Reflection on Key Emerging Concepts from a Reviewed Literature. J Relig Health. 2018 Dec;57(6):2523-2537. doi: 10.1007/s10943-018-0652-9. PMID: 29909518. | 2018 | Spirituality gives purpose to life and life’s experiences, helping one to make meaning of one’s existence, and is inextricably linked to health and well-being . Spirituality is the ‘why’ of life, which helps us build resilience to survive challenges, including health challenges. |
| Pargament, K.I. “The psychology of religion and spirituality? Yes and no,” International Journal for the Psychology of Religion, 1999. vol. 9, no. 1, pp. 3–16. | 1999 | spirituality is “the search for the sacred” |
| Paul Victor CG, Treschuk JV. Critical Literature Review on the Definition Clarity of the Concept of Faith, Religion, and Spirituality. J Holist Nurs. 2020 Mar;38(1):107-113. doi: 10.1177/0898010119895368. Epub 2019 Dec 20. PMID: 31858879. | 2020 | It is an interconnection of something beyond ourselves and connecting something within ourselves , and people interpret and experience their spirituality in different ways through the practice of a certain religion or outside an organized religious system or with a blending of different religious and philosophical traditions . |
| Puchalski C, Ferrell B, Virani R, Otis-Green S, Baird P, Bull J, Chochinov H, Handzo G, Nelson- Becker H, Prince-Paul M, Pugliese K, Sulmasy D: Improving the Quality of Spiritual Care as a Dimension of Palliative Care: The report of the Consensus Conference. J Palliat Med 2009;12:885–904. | 2009 | Puchalski et al ‘‘the aspect of humanity that refers to the way individuals seek and express meaning and purpose and the way they experience their connectedness to the moment, to self, to others, to nature, and to the significant or sacred’’ |
| Puchalski C, Romer AL. Taking a spiritual history allows clinicians to understand patients more fully. J Palliat Med 2000;3:129–37. | 2000 | "spirituality as that which allows a person to experience transcendent meaning in life. This is often expressed as a relationship with God, but it can also be about nature, art, music, family, or community— whatever beliefs and values give a person a sense of meaning and purpose in life. |
| Puchalski CM, Vitillo R, Hull SK, Reller N. Improving the spiritual dimension of whole person care: reaching national and international consensus. J Palliat Med 2014;17: 642e656. | 2012 | Spirituality is a dynamic and intrinsic aspect of humanity through which persons seek ultimate meaning, purpose, and transcendence, and experience relationship to self, family, others, community, society, nature, and the significant or sacred. Spirituality is expressed through beliefs, values, traditions, and practices. International Consensus Conference (2012) |
| Rahimi A, Anoosheh M, Ahmadi F, Foroughan M. Exploring spirituality in Iranian healthy elderly people: A qualitative content analysis. Iran J Nurs Midwifery Res. 2013 Mar;18(2):163-70. PMID: 23983748; PMCID: PMC3748574. | 2013 | Spirituality is the representative of the basic values that guide a person in searching to find answers to the crucial questions of life, such as the purpose and meaning of life, reality, love, good and bad, disease, and death. |
| Rahnama M, Khoshknab MF, Maddah SS, Ahmadi F. Iranian cancer patients' perception of spirituality: a qualitative content analysis study. BMC Nurs. 2012 Oct 9;11:19. doi: 10.1186/1472-6955-11-19. PMID: 23043231; PMCID: PMC3500707. | 2012 | In the present study, relationship with God, having faith and trust in God and obeying God’s orders were defined as spirituality in the view point of the participants. |
| Råholm MB. Weaving the fabric of spirituality as experienced by patients who have undergone a coronary bypass surgery. J Holist Nurs. 2002 Mar;20(1):31-47. doi: 10.1177/089801010202000104. PMID: 11898686. | 2002 | Central aspects of meaning in the concept of spirituality based on this study are spirituality as finding meaning through giving a new appreciation of life and health, spirituality as an inner strength perceived through love and faith, and spirituality as becoming in the dialectic of suffering and desire |
| Ramakrishnan P. 'You are here': locating 'spirituality' on the map of the current medical world. Curr Opin Psychiatry. 2015 Sep;28(5):393-401. doi: 10.1097/YCO.0000000000000180. PMID: 26164614. | 2015 | Our understanding and/or definition of ‘spirituality’ or ‘spiritual care’ may lie in that ‘search’ (for the ‘self’ or the ‘divine’ and the inner dialogue with it) which helps individuals find meaning and purpose in their struggles. |
| Reed PG. An emerging paradigm for the investigation of spirituality in nursing. Res Nurs Health. 1992 Oct;15(5):349-57. doi: 10.1002/nur.4770150505. PMID: 1529119. | 1992 | "A description of spirituality as an expression of the developmental capacity for self-transcendence derives from a developmental-contextual world view of the nature of human beings. Specifically, spirituality refers to the propensity to make meaning through a sense of relatedness to dimensions that transcend the self in such a way that empowers and does not devalue the individual. This relatedness may be experienced intrapersonally (as a connectedness within oneself), interpersonally (in the context of others and the natural environment), and transpersonally (referring to a sense of relatedness to the unseen, God, or power greater than the self and ordinary resources)". |
| Reed PG. Spirituality and well-being in terminally ill hospitalized adults. Res Nurs Health. 1987 Oct;10(5):335-44. doi: 10.1002/nur.4770100507. PMID: 3671781. | 1987 | ‘In terms of personal views and behaviours that express a sense of relatedness to a transcendent dimension or to something greater than the self.’ |
| Relf MV. Illuminating meaning and transforming issues of spirituality in HIV disease and AIDS: an application of Parse's theory of human becoming. Holist Nurs Pract. 1997 Oct;12(1):1-8. doi: 10.1097/00004650-199710000-00003. PMID: 9384065. | 1997 | Spirituality is "broadly defined as (1) belief in and a relationship with a higher power and (2) the aspect of life that gives purpose, meaning, and direction. Spirituality is a transcendental relation­ship with mystery, a "higher being," God, or the universe |
| Renetzky L (1979) The fourth dimension: applications to the social services. In: Moberg D, ed. Spiritual Well Being. University Press of America, Washington: 215–28 | 1979 | ‘…the power within that gives meaning, purpose and fulfilment, the will to live and belief or faith in self, others and in a power beyond self.’ |
| Ross, L., van Leeuwen, R., Baldacchino, D., Giske, T., McSherry, W., Narayanasamy, A., Downes, J., Jarvis, P., Schep-Akkerman, A.,. Student nurses perceptions of spirituality and competence in delivering spiritual care: a European pilot study. Nurse Educ. Today. 2014; 34 (5), 697–702 | 2014 | Definitions available in the literature includes hope and strength; trust; meaning and purpose; forgiveness; beliefand faith in self, others, and for some belief in a deity/higher power;peoples' values; love and relationships; morality; creativity and self-ex-pression (Ross et al., 2014). |
| Russinova Z, Cash D. Personal perspectives about the meaning of religion and spirituality among persons with serious mental illnesses. Psychiatr Rehabil J. 2007 Spring;30(4):271-84. doi: 10.2975/30.4.2007.271.284. PMID: 17458451. | 2007 | DIMENSIONS: Informal, Personal, Exploratory, Continuous character. Personal relationship with the transcendent. Awareness of one’s own soul. Awareness of universal life force. Sense of universal connectedness. |
| Rykkje LL, Eriksson K, Raholm MB. Spirituality and caring in old age and the significance of religion - a hermeneutical study from Norway. Scand J Caring Sci. 2013 Jun;27(2):275-84. doi: 10.1111/j.1471-6712.2012.01028.x. Epub 2012 Jun 24. PMID: 22724432. | 2013 | Connectedness with a Higher power. religiousness cannot be separated from spirituality. |
| Salmon B, Bruick-Sorge C, Beckman SJ, Boxley-Harges S. The evolution of student nurses' concepts of spirituality. Holist Nurs Pract. 2010 Mar-Apr;24(2):73-8. doi: 10.1097/HNP.0b013e3181d39aba. PMID: 20186017. | 2010 | DIMENSIONS/ THEMES: Connectedness to higher power, to others, to self, to God, and to universe/nature. Individual/uniqueness. |
| Schulz EK. Spirituality and disability: an analysis of select themes. Occup Ther Health Care. 2005;18(4):57-83. doi: 10.1080/J003v18n04_05. PMID: 23927653. | 2005 | experiencing a meaningful connection to our core selves, other humans, the world, and/or a greater power as expressed through our reflections, narratives, and actions |
| Seccareccia D, Brown JB. Impact of spirituality on palliative care physicians: personally and professionally. J Palliat Med. 2009 Sep;12(9):805-9. doi: 10.1089/jpm.2009.0038. PMID: 19624268. | 2009 | spirituality as a multidimensional construct that may involve a search for meaning and purpose, a sense of connectedness, a relation to a higher being or power and transcendence |
| Selby D, Seccaraccia D, Huth J, Kurppa K, Fitch M. Patient versus health care provider perspectives on spirituality and spiritual care: the potential to miss the moment. Ann Palliat Med. 2017 Apr;6(2):143-152. doi: 10.21037/apm.2016.12.03. Epub 2017 Jan 8. PMID: 28249545. | 2017 | definition of spirituality with patients highly focused on sensory experiences and living ‘in the moment’ rather than limiting spirituality to belief systems and ‘answers’, connection to a higher being and for many that was indeed an important component of spirituality. This focus on sensory experiences as a core component of spirituality was more prominent in our study |
| Sessanna L, Finnell D, Jezewski MA. Spirituality in nursing and health-related literature: a concept analysis. J Holist Nurs. 2007 Dec;25(4):252-62; discussion 263-4. doi: 10.1177/0898010107303890. PMID: 18029966. | 2007 | THEMES: (a) spirituality as religious systems of = beliefs and values (spirituality religion); (b) spirituality as life meaning, purpose, and connection with others; (c) spirituality as nonreligious systems of beliefs and values; and (d) spirituality as a metaphysical or transcendental phenomena. |
| Sessanna L. The role of spirituality in advance directive decision making among independent community dwelling older adults. J Relig Health. 2008 Mar;47(1):32-44. doi: 10.1007/s10943-007-9144-z. Epub 2007 Sep 11. PMID: 19104999. | 2008 | Spirituality, as noted above, was defined or described as believing in a higher being, as being an innate or intrinsic quality or feeling, and as being good or doing good. |
| Shirahama K, Inoue EM. Spirituality in nursing from a Japanese perspective. Holist Nurs Pract. 2001 Apr;15(3):63-72. doi: 10.1097/00004650-200104000-00011. PMID: 12120113. | 2001 | spirituality was described as “living in harmony with nature and surrounding people. |
| Siddall PJ, Lovell M, MacLeod R. Spirituality: what is its role in pain medicine? Pain Med. 2015 Jan;16(1):51-60. doi: 10.1111/pme.12511. Epub 2014 Aug 26. PMID: 25159525. | 2015 | those aspects of life that lie at the core of a person’s identity and direction, such as the beliefs, values, activities, and relationships that provide meaning and purpose for life |
| Smeltzer, S., Bare, B. (1996). Brunner and Suddarth’s Textbook of Medical–Surgical Nursing. Lippincott Raven Publishers, Philadelphia, PA. | 1996 | Spirituality may be defined as ‘‘personal belief system that focuses on a search for meaning and purpose in life ... and a connectedness to a higher dimension’’. |
| Smith J, McSherry W. Spirituality and child development: a concept analysis. J Adv Nurs. 2004 Feb;45(3):307-15. doi: 10.1046/j.1365-2648.2003.02891.x. PMID: 14720248. | 2004 | spirituality is concerned with existentialism, connectedness or interconnectedness within oneself, other people and the universe at large, and that it comes into focus during times of crisis. |
| Smith S. Toward a flexible framework for understanding spirituality. Occup Ther Health Care. 2008;22(1):39-54. doi: 10.1080/J003v22n01_04. PMID: 23944762. | 2008 | spirituality is central to a person and is interpreted through his/her personal worldview. These two concepts, center and personal worldview, when related in this way, may assist each person to identify and express his/her particular understanding of spirituality. |
| Smyth T, Allen S. Nurses' experiences assessing the spirituality of terminally ill patients in acute clinical practice. Int J Palliat Nurs. 2011 Jul;17(7):337-43. doi: 10.12968/ijpn.2011.17.7.337. PMID: 21841702. | 2011 | Their understanding of spirituality as a personal journey, their feelings that spirituality may or may not be linked to religion |
| Solomon R. 2002. Spirituality for the skeptic: The thoughtful love of life. New York: Oxford Univ. Press. | 2002 | Spirituality describes a mode of being-in-the-world that provides an avenue for transcendent meaning, and categories for understanding the major events in our lives. This may have little to do with organized religious practices, or formal doctrine, and is frequently grounded in personal experiences and convictions rather than ecclesiastical authorities. |
| Stephenson PS, Berry DM. Describing Spirituality at the End of Life. West J Nurs Res. 2015 Sep;37(9):1229-47. doi: 10.1177/0193945914535509. Epub 2014 May 25. PMID: 24862937. | 2015 | five attributes that most commonly described the essence of spirituality, including meaning, beliefs, connecting, self-transcendence, and value. |
| Stoll RI. (1989) The essence of spirituality. ln: Carson VB, ed. Spiritual Dimensions of Nursing Practice. Phila- delphia: Saunders; 1989. | 1989 | Spirituality is "a way of being and experiencing that comes about through awareness of a transcendent dimension characterized by certain identifiable values in regard to self, others, nature, life, and whatever one considers the "Ultimate". |
| Surbone, A., Konishi, T., & Baider, L. (2011). Spiritual issues in supportive cancer care. In I. N. Oliver (Ed.), The MASCC textbook of cancer supportive care (pp. 419-425). New York, NY: Springer | 2011 | Five central features of spirituality: (a) meaning, the significance of life, making sense of life, and deriving purpose in existence; (b) value, cherished beliefs, and standards, having to do with beauty, truth, and worth; (c) transcendence, experience, and appreciation of dimension beyond one’s self; (d) connecting relationships with self, others, God/higher power, and the environment; and (e) becoming, the unfolding of life that demands reflection and experience, including a sense of who one is and how one knows. |
| Swinton J, Pattison S. Moving beyond clarity: towards a thin, vague, and useful understanding of spirituality in nursing care. Nurs Philos. 2010 Oct;11(4):226-37. doi: 10.1111/j.1466-769X.2010.00450.x. PMID: 20840134. | 2010 | Spirituality is related to issues of meaning, hope, purpose, connectedness, love, and so forth, the implication being that these things are perceived as missing or downplayed within current approaches to care and treatment |
| Tanyi RA. Towards clarification of the meaning of spirituality. J Adv Nurs. 2002 Sep;39(5):500-9. doi: 10.1046/j.1365-2648.2002.02315.x. PMID: 12175360. | 2002 | Spirituality is a personal search for meaning and purpose in life, which may or may not be related to religion. It entails connection to self-chosen and or religious beliefs, values, and practices that give meaning to life, thereby inspiring and motivating individuals to achieve their optimal being. This connection brings faith, hope, peace, and empowerment. The results are joy, forgiveness of oneself and others, awareness and acceptance of hardship and mortality, a heightened sense of physical and emotional well-being, and the ability to transcend beyond the infirmities of existence. |
| Timmins F, Caldeira S. Understanding spirituality and spiritual care in nursing. Nurs Stand. 2017 Jan 25;31(22):50-57. doi: 10.7748/ns.2017.e10311. PMID: 28120672. | 2017 | spirituality is the notion that people strive to make sense of, and derive meaning from, life events and seek to connect with the self, others and their community. This sense of meaning and connection is underpinned by a personal belief system that may be informed by religious beliefs. |
| Torskenæs KB, Baldacchino DR, Kalfoss M, Baldacchino T, Borg J, Falzon M, Grima K. Nurses' and caregivers' definition of spirituality from the Christian perspective: a comparative study between Malta and Norway. J Nurs Manag. 2015 Jan;23(1):39-53. doi: 10.1111/jonm.12080. Epub 2013 Jul 4. PMID: 23822866. | 2015 | Spirituality was considered as an internal energy, larger than the self and associated with the environment. spirituality was considered as a resource of inspiration, connectedness and energy to be altruistic in life. Spirituality was viewed in a wider perspective which may penetrate everything and every person universally. the Maltese groups identified first the religious perspective of spirituality and emphasized the connectedness with self, others/family, nature and God/higher power. |
| Tuck I, Thinganjana W. An exploration of the meaning of spirituality voiced by persons living with HIV disease and healthy adults. Issues Ment Health Nurs. 2007 Feb;28(2):151-66. doi: 10.1080/01612840601096552. PMID: 17365165; PMCID: PMC2211367. | 2007 | Themes for Persons Living with HIV Disease: Spirituality is Relating, and Believing in God or a Higher Power/ Spirituality is Being Guided or Helped/ is Being Inspired by or Receiving Gifts/ Spirituality is Expressed in Outward Ways /Spirituality is Journeying, Discovering, Centering/ is Feeling the Presence of God - THEMES FOR healthy adults yielded six themes: a belief in a personal relationship with God; a connection and relationship with others; a spiritual journey, guide, or struggle; the spiritual essence of self; spirituality expressed in actions; and integral spirituality. |
| Unruh AM, Versnel J, Kerr N. Spirituality unplugged: a review of commonalities and contentions, and a resolution. Can J Occup Ther. 2002 Feb;69(1):5-19. doi: 10.1177/000841740206900101. PMID: 11852691. | 2002 | The themes are: 1) relationship to God, a spiritual being, a higher power, or a reality greater than the self; 2) not of the self; 3) transcendence or connectedness unrelated to a belief in a higher being; 4) existential, not of the material world; 5) meaning and purpose in life; 6) life force of the person, integrating aspect of the person; and 7) summative. |
| Utsch M. Spiritualität – Wert der Beziehung [Spirituality - Value of the relationship]. Nervenarzt. 2016 Nov;87(11):1152-1162. German. doi: 10.1007/s00115-016-0228-4. PMID: 27752722. | 2016 | DIMENSIONS: Search for meaning and ability to Self-transcendence, Connection with a higher one Power, self-acceptance and self-developmenttion, positive social relationships,intense experience of beauty or Holiness of nature, mindfulness or other meditation on experiences |
| Vachon M, Fillion L, Achille M. A conceptual analysis of spirituality at the end of life. J Palliat Med. 2009 Jan;12(1):53-9. doi: 10.1089/jpm.2008.0189. PMID: 19284263. | 2009 | “developmental and conscious process, characterized by two movements of transcendence; either deep within the self or beyond the self.” |
| Vachon ML. Meaning, spirituality, and wellness in cancer survivors. Semin Oncol Nurs. 2008 Aug;24(3):218-25. doi: 10.1016/j.soncn.2008.05.010. PMID: 18687268. | 2008 | Spirituality is a construct composed of faith and meaning,5 an attempt to make contact with or become aware of the ‘‘deep knowing’’ of our being. |
| Villagomeza LR. Mending broken hearts: the role of spirituality in cardiac illness: a research synthesis, 1991-2004. Holist Nurs Pract. 2006 Jul-Aug;20(4):169-86. doi: 10.1097/00004650-200607000-00004. PMID: 16825919. | 2006 | DIMENSIONS: 1 Sense of connectedness: intrapersonal, personal, ecological, and transpersonal connectedness. Relationships within the realms of self, others,a higher power, and nature. 2. Faith and religious belief system: Belief in God or higher power, Belief in afterlife, Faith, Prayer, Religion and religiosity. 3. Value system: cherished standards, Positive attitude. 4. Sense of meaning and purpose: existential meaning, Finding meaning in suffering, Enduring illness, Essence of being, Facing mortality. 5. Sense of self-transcendence: expansion of personal boundaries, Becoming, Forgiveness, Love, 6. Sense of inner peace and harmony: harmonious state of mind, Acceptance, Comfort, Letting go of fear, Peace, Well-being. 7. Sense of inner strength and energy: vital life force, Courage, Energy, Hope, Inner strength, Life-giving force, Motivating force, Optimism , Will to live |
| Walach, H. , Kohls, N. , Von Stillfried, N. , Hinterberger, T. & Schmidt, S . Spirituality: The legacy of parapsychology . Archive for the Psychology of Religion . 2009; 31 , 277 – 308 . | 2009 | spirituality has as a common definitional core some experiential, notional, behavioral or intentional relationship with some transcendent reality, out of which arises meaning, solace or motivation for an individual. (...) we could bring the pieces together in a brief factual description of what spirituality means an increase in non-local connectedness between an individual and the totality, and by the very same token also between the individual and its own subsystems and other individuals. |
| Walsh, R. (1999). Essential spirituality. The 7 Central Practices to Awaken Heart and Mind. New York: John Wiley | 1999 | the primary energy center at which the transcendent or divine dimension of existence is encountered, and as a set of beliefs and practices relative to transformation of self. |
| Walton J. Spirituality of patients recovering from an acute myocardial infarction. A grounded theory study. J Holist Nurs. 1999 Mar;17(1):34-53. doi: 10.1177/089801019901700104. PMID: 10373841. | 1999 | Spirituality was a life-giving force that came from within each participant. This life-giving force called spirituality was nurtured by receiving presence from God, nature, friends, family, and community, and was based on developing faith, discovering meaning and purpose, and giving the gift of self. |
| Weathers E, McCarthy G, Coffey A. Concept Analysis of Spirituality: An Evolutionary Approach. Nurs Forum. 2016 Apr;51(2):79-96. doi: 10.1111/nuf.12128. Epub 2015 Feb 2. PMID: 25644366. | 2016 | Spirituality is a way of being in the world in which a person feels a sense of connectedness to self, others, and/or a higher power or nature; a sense of meaning in life; and transcendence beyond self, everyday living, and suffering. |
| Wein S. Spirituality--the psyche or the soul? Palliat Support Care. 2014 Apr;12(2):91-4. doi: 10.1017/S1478951514000303. PMID: 24635943. | 2014 | secular “spirituality” is constituted by an experience of unification accompanied by a change in the state of consciousness. |
| White ML, Peters R, Schim SM. Spirituality and spiritual self-care: expanding self-care deficit nursing theory. Nurs Sci Q. 2011 Jan;24(1):48-56. doi: 10.1177/0894318410389059. PMID: 21220576. | 2011 | Spirituality is defined as the beliefs a person holds related to a subjective sense of existential connectedness including beliefs that reflect relationships with others, acknowledge a higher power, recognize an individual’s place in the world, and lead to spiritual practices. |
| Worthington EL Jr, Hook JN, Davis DE, McDaniel MA. Religion and spirituality. J Clin Psychol. 2011 Feb;67(2):204-14. doi: 10.1002/jclp.20760. PMID: 21108313. | 2011 | Spirituality, in contrast, can be defined as a more general feeling of closeness and connectedness to the sacred. What one views as sacred is often a socially influenced perception of either (a) a divine being or object or (b) a sense of ultimate reality or truth. Many people experience their spirituality in the context of religion, but not all do. |
| Yang CT, Narayanasamy A, Chang SL. Transcultural spirituality: the spiritual journey of hospitalized patients with schizophrenia in Taiwan. J Adv Nurs. 2012 Feb;68(2):358-67. doi: 10.1111/j.1365-2648.2011.05747.x. Epub 2011 Jun 24. PMID: 21707724. | 2012 | To clarify spirituality in the context of Taiwanese culture, an explication of spirituality as lived experience follows. In Taiwanese, and more widely, in Chinese culture, there is more than one noun to represent the same concept of the Western definition of spirituality: a mixture of Confucianism, Taoism or folk beliefs provide guidance on how to be a human being with a meaningful life with principles such as (Middle way), (practice charity), (practice filial piety), exploring harmony and life meaning through (the cardinal human relations), balancing external environment (universal self) with the inner environment (small self, ) and achieving – (the universe, nature, body and soul of self-communion as a whole integer and holistic transcendence) |
| Yawar A. Spirituality in medicine: what is to be done? Journal of Research and Social Medicine 2001; 94: 529–532. | 2006 | In discussing spirituality, one is really discussing the ways in which people fulfil what they hold to be the purpose of their lives. (...) Evident that there are as many spiritualities as there are human beings. (...) Human beings may be considered to have two realms of existence, the outer and inner realms. The outer realm consists of a person’s interaction with the world, whereas the inner realm has been defined as the individual’s interaction with the transcendental. This may be a divine being or ideals hinted at through experiences such as beauty, awe and love. |
